# Allosteric Regulation of Photophysics and Binding in Oxazine‐macrocycle Complexes at Single‐molecule Resolution

**DOI:** 10.1002/anie.1546846

**Published:** 2026-05-31

**Authors:** Siyu Lu, Thomas‐Otavio Peulen, Hongbin Wu, Florian Lindemann, Despoina Kapiki, Rasmus Linser, Andreas Hennig, Thorben Cordes

**Affiliations:** ^1^ Biophysical Chemistry Department of Chemistry and Chemical Biology Technische Universität Dortmund Dortmund Germany; ^2^ Physical and Synthetic Biology Faculty of Biology Ludwig‐Maximilians‐Universität München Planegg‐Martinsried Germany; ^3^ Biomolecular NMR Spectroscopy Department of Chemistry and Chemical Biology Technische Universität Dortmund Dortmund Germany; ^4^ Center For Cellular Nanoanalytics (CellNanOs) and School of Biology / Chemistry Universität Osnabrück Osnabrück Germany

**Keywords:** allostery, calixarene, cucurbituril, host‐guest chemistry, oxazine fluorophores, photochemistry, photophysics, single‐molecule spectroscopy, supramolecular chemistry

## Abstract

Dye‐macrocycle complexes have become invaluable tools for various applications. The mechanisms of complex formation and photophysical changes can be complex and difficult to characterize. This is due to limited information available from bulk experiments on heterogeneity, complex stoichiometry, kinetic rates, and a distinction of photophysical effects caused by complex formation or collisional processes. Recent advances in the use of single‐molecule spectroscopy have begun to bridge this gap. Here, we investigate the interactions between the oxazine fluorophore ATTO655 and two macrocyclic hosts, cucurbit[8]uril (CB8) and *p*‐sulfonatocalix[4]arene (sCX4). Although ATTO655‐CB8 shows the classical picture of an inclusion complex, the interactions of ATTO655 with sCX4 are largely unprecedented. ATTO655 forms dim exclusion complexes via external binding to the aromatic moeities of sCX4. Here, the upper portal and hydrophobic cavity of sCX4 remain accessible for known guests such as the neurotransmitter choline. In this ternary complex, choline reduces the binding affinity and prevents the formation of photoinduced dark states observed in single‐molecule traces, which presents a form of allosteric regulation of binding and photophysics. Our study is a showcase for the power of single‐molecule spectroscopy to unravel fundamental mechanisms of host–guest systems, which can facilitate their use in advanced bioimaging and sensing applications.

## Graphical abstract

This work explores the interactions between the oxazine dye ATTO655 and two macrocyclic hosts using optical (single‑molecule) spectroscopy. Although ATTO655 forms a classical inclusion complex with cucurbit[8]uril (CB8), its interaction with p‑sulfonatocalix[4]arene (sCX4) leads to the formation of dim exclusion complexes. Here, the cavity and upper portal of sCX4 remain accessible to secondary guests such as choline, enabling the formation of a ternary complex. Choline modulates the binding affinity and suppresses photoinduced dark states, revealing an allosteric regulation of binding and photophysics. These results highlight the ability of single‑molecule spectroscopy to resolve complex host–guest mechanisms relevant for bioimaging and sensing applications.

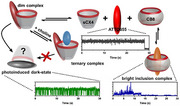

## 1 Introduction

Macrocycles are ring‐shaped molecules that form supramolecular complexes with various binding partners including (fluorescent) dyes. The complexes are formed via specific noncovalent interactions between the dye and the macrocycle, which hosts the dye at its portals or encapsulates it within an inner cavity. Since such interactions often result in characteristic optical changes, including color‐, lifetime‐, or intensity changes, the formed complexes are powerful tools for applications in chemistry and biology [1, 2] such as molecular sensing [3], kinetic assays [4, 5, 6], imaging [1, 7, 8, 9], phototheranostics [10, 11, 12], or photonic materials [13]. A selection of relevant macrocycles for this includes cyclodextrins [2, 5, 14, 15], calixarenes [16, 17, 18], other cyclophanes [9, 11, 13], and cucurbiturils [8, 19, 20, 21], which undergo binding with dyes from various structural classes [22, 23], and numerous other guest molecules [24, 25].

Despite this success in many different research fields, it is striking and has been highlighted recently [26] that the characterization of host–guest chemistry is so far done almost exclusively in bulk experiments, e.g., via nuclear magnetic resonance (NMR), isothermal titration calorimetry (ITC), transient absorption spectroscopy [13] and UV–vis and fluorescence spectroscopy [16, 19]. Consequently, there are many detailed studies on the spectroscopic properties of dye‐macrocycle complexes [15, 19, 21, 27, 28, 29], yet single‐molecule fluorescence spectroscopy has only been used in a handful of these [5, 20, 26]. This approach can, however, reveal important features of static and dynamic heterogeneity in host–guest systems, visualize complex stoichiometry, and provide access to the kinetics of complex association and dissociation in a straightforward manner. The most recent examples are the use of single‐molecule video imaging to determine the stoichiometry of supramolecular complexes [26], host–guest chemistry to improve dye photostability and brightness [1, 30] and to generate fluorogenic probes for single‐molecule localization microscopy [31].

**FIGURE 1:**
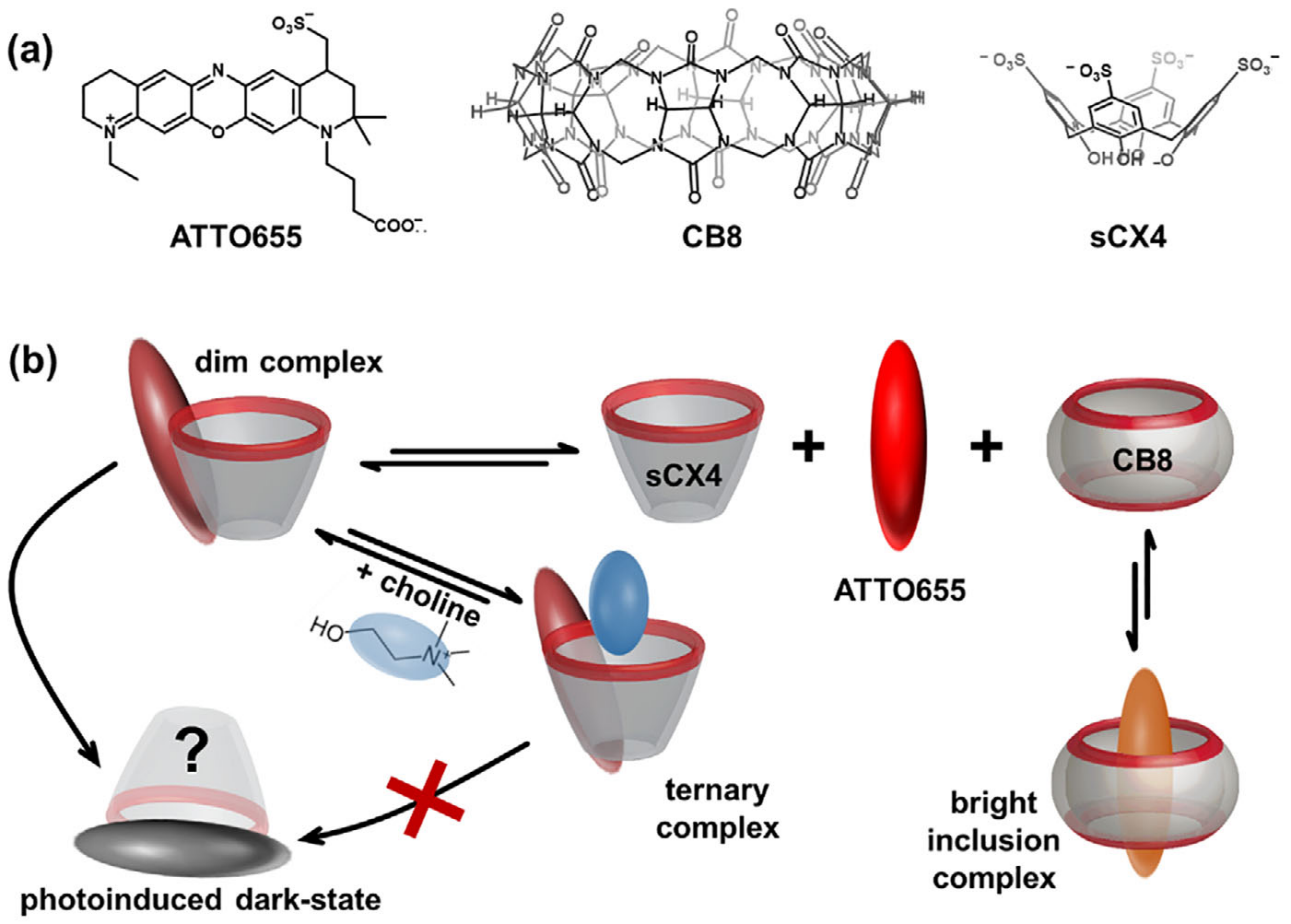
(a) Chemical structures and (b) schematics of the proposed interactions and photophysical properties of the oxazine dye ATTO655 and the macrocycles used in this study.

We here present a single‐molecule fluorescence spectroscopy study of the oxazine fluorophore ATTO655 in complex with two distinct macrocycles: cucurbit[8]uril (CB8) and *p*‐sulfononatocalix[4]arene (sCX4); Figure 1a. Oxazines are a popular choice for biological single‐molecule spectroscopy [32, 33, 34, 35, 36] and super‐resolution microscopy [37, 38, 39, 40, 41, 42] due to their high photostability, particularly under oxygenated conditions in aqueous buffer [42], which is otherwise challenging and requires e.g., triple‐state quenchers for optimal performance [43]. Both selected macrocycles are considered classical cation receptors. CB8 has glycoluril portals with partial negative charges, enabling strong ion‑dipole and hydrogen‑bonding interactions with cationic guests. Its interior is a nonpolar, methylene‑bridged and cylindrical cavity (∼8.8 Å diameter) that preferentially hosts non‐polar fragments [44]. sCX4 has a negatively charged upper rim of sulfonate‑groups enabling electrostatic interactions and a lower rim of phenolic hydroxy groups that is stabilized by hydrogen bonding. Its binding cavity (∼3.8 Å diameter [45]) is formed by the nonpolar, aromatic parts of the phenol units and can bind aromatic or aliphatic fragments by hydrophobic interactions and cations by cation‐π interactions.

Using a combination of bulk absorption and fluorescence spectroscopy with fluorescence correlation spectroscopy (FCS), time‐correlated single‐photon counting (TCSPC) and single‐molecule detection, we provide a detailed view on the molecular interactions between the dye and both macrocycles and the photophysical consequences. Although ATTO655‐CB8 shows the characteristics of a classical inclusion complex, the interactions of ATTO655 with sCX4 are largely unprecedented (Figure 1b). sCX4 is a classical cation‐receptor, yet it binds the anionic ATTO655 with moderate affinity (*K*_a_ ∼2 × 10^3^ M^−1^). Detailed analysis of optical spectroscopic data, NMR experiments and structural modelling suggests that ATTO655 is not internalized into the hydrophobic cavity and does also not block the upper negatively‐charged portal, but instead forms an exclusion complex binding the aromatic sCX4 moeities externally. This allows for the formation of ternary complexes with known sCX4 guests such as the neurotransmitter choline at the top of the cavity (Figure 1b). Strikingly, the new binding mode enables an allosteric regulation of the sCX4 binding affinity to ATTO655 and of a photoinduced reaction between them, which we were able to track at single‐molecule resolution (Figure 1b).

The present study is a showcase for the ability of single‐molecule spectroscopy to unravel fundamental new mechanisms in host‐guest systems. Our discovery provides a blueprint for the future use of dye‐macrocycle complexes in advanced bioimaging and sensing applications by controlling the degree of fluorescence quenching or enhancement, binding affinity and stoichiometry, and the kinetics of binding and host‐induced (chemical) reactions.

## 2 Results and Discussion

### 2.1 Optical Characterization of the CB8‐ATTO655 Complex

**FIGURE 2:**
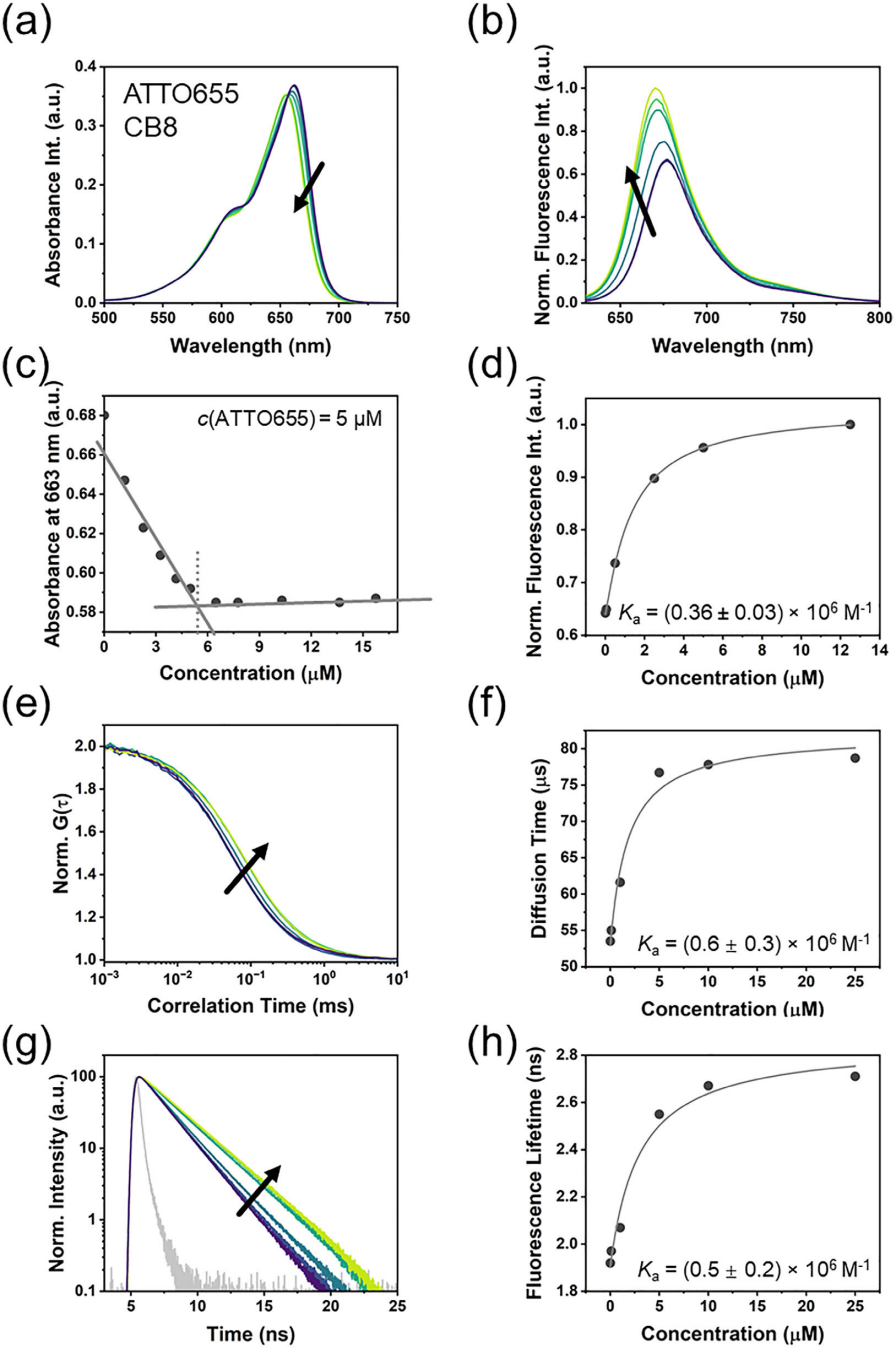
Spectroscopic analysis of the interaction between ATTO655 and CB8 (0–25 µM, color gradient from dark to light indicates increasing concentration). (a) Absorbance and (b) fluorescence emission spectra (619 nm excitation) at varying CB8 concentrations. The specific concentrations are provided in the titration panels (d, f, h), where the spectroscopic parameters are shown as a function of macrocycle concentration [M_0_]. (c) Absorbance titration of ATTO655 (5 µM) with CB8, where the crossing point of two linear interpolations at 5.2 µM is the concentration of CB8 required for full complexation of ATTO655. (d) Fluorescence titration with normalized fluorescence intensities and the results of a fit to obtain *K*_a_. (e) Fluorescence correlation spectroscopy and (f) the corresponding diffusion times as a function of CB8 concentration. (g) Normalized TCSPC decays and (h) the corresponding mean fluorescence lifetimes, where grey lines represent fit functions. Experimental conditions for (e–h): 640 nm excitation with 25 µW (8.5 kW cm^−2^) using an oil objective (details in the methods); *n* = 3. All experiments were performed in 5 mM KP_i_ at pH 7.4.

We found that the addition of CB8 to ATTO655 in aqueous buffer shows clear spectroscopic signatures of complex formation and fluorophore inclusion (Figure 2). Absorption spectra (Figure 2a) showed a hypsochromic shift of 7 nm and a decreased absorption with excess CB8, and fluorescence spectra (Figures 2b, S1) showed an increase in emission intensity by 39% and a hypsochromic shift of 7 nm. The excitation in our fluorescence experiment was performed at the isosbestic point at 619 nm, ensuring that changes in the overall emission intensity were not caused by changes of the extinction coefficient. Consequently, the fluorescence quantum yield of ATTO655 increases in accordance with the fluorescence intensity. The spectral signatures resemble those known for Oxazine 1 in complex with cucurbit[7]uril (CB7) and CB8 [19], but also those for ATTO655 [1], ATTO680, and ATTO700 [30] in complex with CB7.

The respective absorption titration (Figure 2c) indicated strong 1:1 binding and for a fluorescence titration gave access to the association equilibrium constant (*K*_a_) of (0.36 ± 0.03) × 10^6^ M^−1^ and the dissociation equilibrium constant (*K*_d_) of 2.78 µM (calculated as 1/*K*_a_) from nonlinear least‐square fitting with a simple and an accurate 1:1 binding model (Figure 2d, S2). These results are in agreement with results from the ATTO655‐CB7 complex, but also that of other dyes, such as Oxazine 1, in complex with CB7 and CB8 [19], which are known to be primarily 1:1 complexes. Other key spectroscopic parameters are summarized in Table 1.

**TABLE 1:** Photophysical properties of ATTO655 in the absence and presence of macrocyclic hosts (25 µM CB8 or 10 mM sCX4) in KP_i_ buffer at pH 7.4. The listed parameters include the absorbance (*λ*_abs_) and emission (*λ*_fl_) maxima, the relative fluorescence intensity (*F*/*F*_0_), diffusion times (*τ*_d_), amplitude‐weighted mean fluorescence lifetimes (〈*τ*〉_Amp._), and association equilibrium constant (*K*_a_) derived from fluorescence titrations.

Condition	*λ*_abs_ (nm)	*λ*_fl_ (nm)	Rel. Fl. (*F*/*F*_0_)	*τ*_D_ (µs)	〈*τ*〉_Amp._ (ns)	*K*_a_ (M^−1^)
Free ATTO655	662	677	1	55 ± 1	1.88 ± 0.04	N.A.
+ CB8	655	670	1.48 ± 0.06	82 ± 4	2.71 ± 0.11	(0.36 ± 0.03) × 10^6^
+ sCX4	661	678	0.234 ± 0.004	80 ± 3	0.44 ± 0.08	(2.11 ± 0.14) × 10^3^

To confirm complex formation via an orthogonal method, we used fluorescence correlation spectroscopy (FCS) to obtain information on the size, molecular brightness, and blinking kinetics of ATTO655 both in its free and macrocycle‐bound state. We consider this an important addition, particularly in cases in which the relation of spectral changes to complex formation are less clear or need to be separated from possible collisional processes, i.e., when the macrocycle has lower affinity. For free ATTO655, the molecular brightness was ∼29 kHz at 25 µW excitation power (8.5 kW cm^−2^ power density), while the diffusion time (*τ*_d_) was 55 ± 1 µs (Figures 2e, *n* = 4). As for other FCS studies of ATTO655, the triplet‐related bunching amplitude was low and barely observable at low excitation powers (Figure S3). In agreement with the spectral data in Figure 2, the brightness and most importantly the diffusion time of ATTO655 increased upon addition of CB8 in a concentration dependent manner from 55 to 82 µs (Figure 2e,f; Table 1, Figure S3). Considering the published diffusion coefficient of ATTO655 of 4.26 × 10^−6^ cm^2^ s^−1^ [46], this corresponds to an increase of the hydrodynamic radius *R*_h_ from about ∼0.58 to ∼0.86 nm. A fit to the plot of the diffusion time of ATTO655 as a function of the concentration of CB8 gives a *K*_a_ of (0.6 ± 0.3) × 10^6^ M^−1^ (Figure 2f), which is consistent with the fluorescence changes and directly links these to complex formation on a molecular level via two orthogonal methods.

**FIGURE 3:**
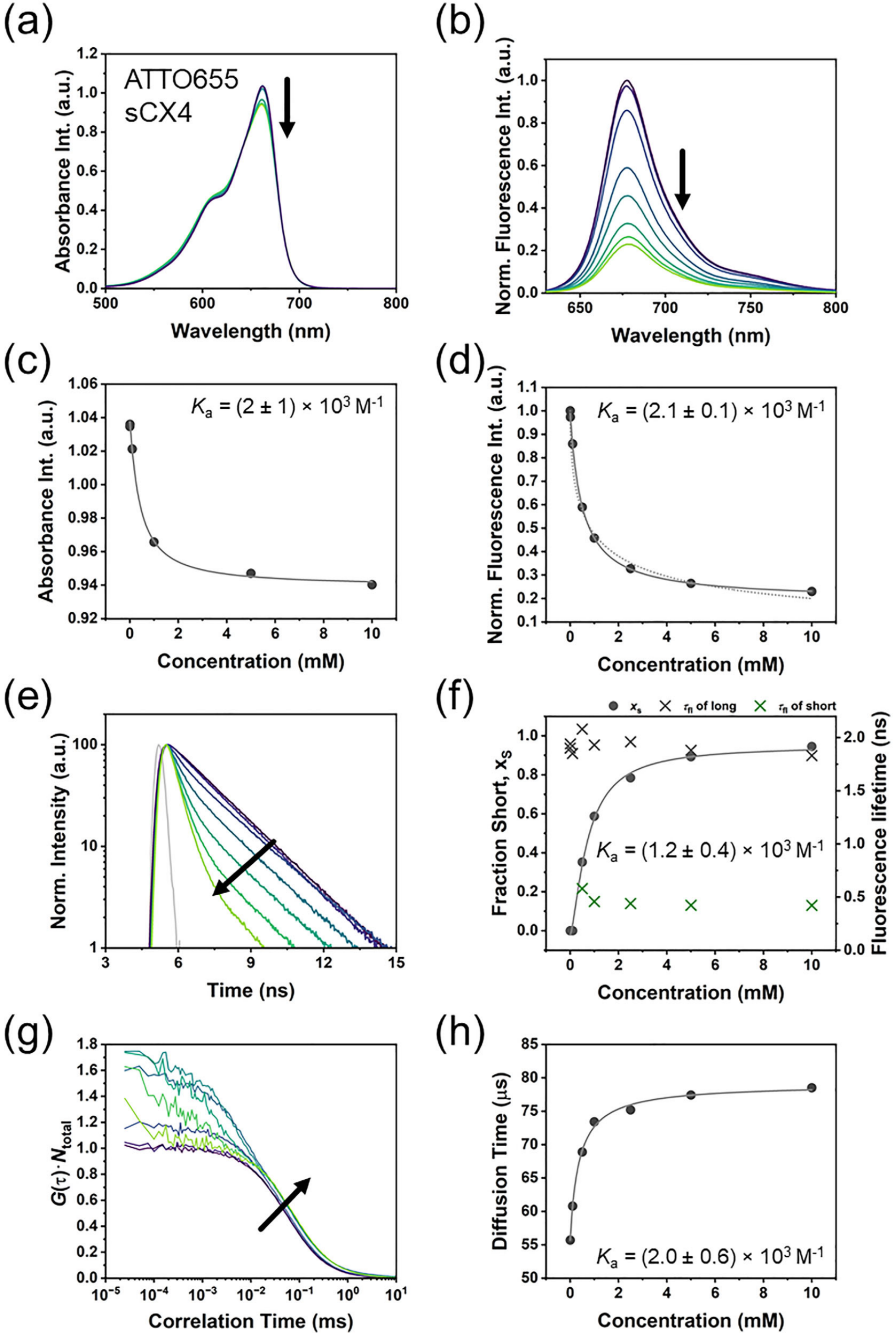
Spectroscopic and microscopic analysis of the interaction between ATTO655 and sCX4 (0–10 mM, color gradient from dark to light indicates increasing concentration). (a) Absorbance and (b) fluorescence spectra (620 nm excitation) with different concentration of sCX4. (c) Absorbance and (d) normalized fluorescence titration of ATTO655 with sCX4 and the results of fits (grey line). The grey line in (d) indicates the fit that with a fixed endpoint at a normalized fluorescence of 0. (e) Normalized TCSPC curves and (f) corresponding fraction of short lifetime component, fluorescence lifetime of short and long components at varying concentrations of sCX4. (g) Results from FCS experiments of ATTO655 with sCX4 and (h) corresponding fit. Experimental conditions for (e–h): 640 nm excitation, 25 µW (8.5 kW cm^−2^), oil objective. All experiments were performed in 100 mM KP_i_, pH 7.4.

Finally, we conducted time‐resolved fluorescence measurements via time‐correlated single‐photon counting (TCSPC). A single exponential deconvolution fit revealed an increase of the fluorescence lifetime of ATTO655 from 1.88 ± 0.04 to 2.71 ± 0.11 ns upon complex formation (Figure 3g). A fit of the concentration dependence using the mean fluorescence lifetime provides a *K*_a_ of (0.5 ± 0.2) × 10^6^ M^−1^ (Figure 3h), which is again consistent with the other experiments. Based on the combined data, complex formation between ATTO655 and CB8 was conclusively validated due to the observed increase in molecular size via FCS, which is accompanied by spectral and lifetime changes, that are all compatible with encapsulation of the dye.

### 2.2 Optical Characterization of the sCX4‐ATTO655 Complex

Using the same experimental strategy, we studied the interaction of ATTO655 with a distinct macrocycle (sCX4). Based on literature data, we expected a static quenching mechanism for sCX4‐binding possibly involving photoinduced electron transfer (PET) [47]. In line with this idea, the addition of 10 mM sCX4 to ATTO655 in aqueous buffer resulted in strong fluorescence quenching by 77% with hardly any spectral shifts (Figure 3a,b). Nonlinear fitting with a 1:1 binding model of the respective titration plots (Figure 3c,d) gave *K*_a_ of (2 ± 1) × 10^3^ M^−1^ (*K*_d_ = 0.5 mM) from the absorbance titration and a *K*_a_ of (2.1 ± 0.1) × 10^3^ M^−1^ (*K*_d_ = 0.48 mM) from a fluorescence titration. Importantly, isothermal titration calorimetry (ITC) verifies binding and shows a *K*_a_ value of (1.00 ± 0.07) × 10^3^ M^−1^ (*K*_d_  = 1.00 mM) with a 1:1 stoichiometry (*n* = 3, Figure S4).

In contrast to our expectation, we noticed that the fluorescence intensity does not reduce to baseline values even at high concentrations, and that the absence of spectral shifts particularly in the absorbance spectrum is distinct compared to other dye molecules such as lucigenin (LCG) [47]. Furthermore, classical Stern–Volmer analysis of the fluorescence data revealed a nonlinear behavior in the form of a downward curvature. Such behavior can be explained by incomplete quenching in which the “quenched” dim complex shows residual fluorescence [48, 49]. In line with this, we found strong changes in the ATTO655 TCSPC histogram, which changes from a mono‐to a bi‐exponential decay when sCX4 is present (Figure 3e). At concentrations >0.1 mM, we find a long lifetime component, which is identical to the free unbound dye (∼1.90 ns) together with a short lifetime, which we attribute to the bound complex (∼0.45 ns; Figure 3f). This is compatible with the proposed static quenching mechanism, in which the bound complex is not fully quenched, since we observe two constant lifetime values above the instrument response function (IRF) of our system. Plotting the fraction of either short‐ or long‐lifetime component as a function of sCX4 concentration and fiting them to a 1:1 binding model provides a *K*_a_ value of (1.2 ± 0.4) × 10^3^ M^−1^ (*n* = 3; Figure 3f). Furthermore, the observed decrease in mean fluorescence lifetime (Figure S5) as a function of sCX4 concentration is consistent with the intensity quenching seen in the steady‐state fluorescence spectra (Figure 3b). Collision‐based dynamic quenching between ATTO655 and sCX4 becomes relevant to a small extent at [sCX4] > 1 mM, seen in a subtle reduction in the lifetime value of the long fluorescent decay component (Figure 3f).

The formation of an ATTO655‐sCX4 complex was then further validated by FCS experiments, which showed a consistent 4‐fold reduction of molecular brightness (Figure S6) and an increase of diffusion times from 55 to 80 µs (Figure 3g,h). This corresponds to an increase of *R*_h_ from ∼0.58 to ∼0.84 nm [46]. Since these findings of quenching and slower diffusion were consistent with all other data (Figure 3), we were able to obtain an internally consistent *K*_a_ value for binding of (2.0 ± 0.6) × 10^3^ M^−1^ (*n* = 3; Figure 3h). Importantly, we observe an additional bunching term on timescales < 10 µs, which is maximal for concentrations around the *K*_d_ of ∼0.5 mM (Figure S7). Based on previous work on cyclodextrin‐complexes [5], the relaxation time we found (4.1 µs) can be attributed to sum of rates for complex formation and dissociation in equilibrium. This interpretation is also supported by additional experiments as discussed below.

### 2.3 Binding Modes of the Dye‐Macrocycle Complexes

Host–guest complexation of dyes (Figure 1) impacts their photophysical behavior due to an altered physico‐chemical environment, which is typically assigned to changes in the solvent shell, i.e., the solvent polarity and polarizability. Furthermore, complexation affects quenching by the solvent or introduces new quenching pathways by the macrocycle [27]. In the following, we demonstrate how a careful interpretation of all of the above spectroscopic data can be used to elucidate the binding modes of host–guest complexes between ATTO655 with CB8 and sCX4.

With respect to cucurbiturils, the inner cavity is known as a low‐polarity region, while the portal regions are highly polar (Figure 1) [25]. The Nau group further described the cavity of cucurbiturils as a “gas‐phase‐like” environment with a polarizability of the CB7 cavity that is even lower than that of perfluorohexane, a solvent known for its low polarizability [27]. The fact that both, absorbance and emission maxima, show hypsochromic shifts (Figure 2a,b) upon complexation is well explained by dye‐relocation into an environment of low polarity, namely binding of the dye into the host cavity. This interpretation is fully compatible with ATTO655 encapsulation into CB8 based on FCS (Figure 2e,f). We further found that both, fluorescence quantum yield *ϕ*_fl_ and lifetime *τ*_fl_, increase 1.48‐fold and 1.44‐fold, respectively, upon binding to CB8. According to the Strickler–Berg equation, the low polarizability would lead to a decreased radiative lifetime, *k*_Fl_, such that a lifetime increase and concomitant quantum yield decrease is expected [27]. Since both values show a similar increase we can safely exclude changes of *k*_Fl_ > 5% and attribute changes of both parameters to the fact that encapsulation of the dye reduces water‐quenching [50]. Inclusion complex formation would cause a partial replacement of the water shell by the macrocycle cavity and we estimate that ∼40% of the dye surface remains water‐accessible, while ∼60% are in a water‐occluded environment of the cavity [15, 51].

Luckily, our data from ITC and FCS strongly support the formation of an ATTO655‐sCX4 complex. This complex is, however, of distinct nature compared to ATTO655‐CB8 since no spectral shifts are observed (Figure 3a,b). Instead ATTO655 experiences strong fluorescence quenching caused by the formation of a nearly dim ATTO655‐sCX4 complex with a lifetime of ∼0.5 ns. Considering the fact that both cyclodextrins and calixarenes provide more polar cavities with polarizabilities that are higher than for cucurbiturils [27], we would have expected (bathochromic) spectral shifts for a true encapsulation of ATTO655 into sCX4. Their absence renders even a partial encapsulation of the central aromatic moieties of ATTO655 into the cavity unlikely.

**FIGURE 4:**
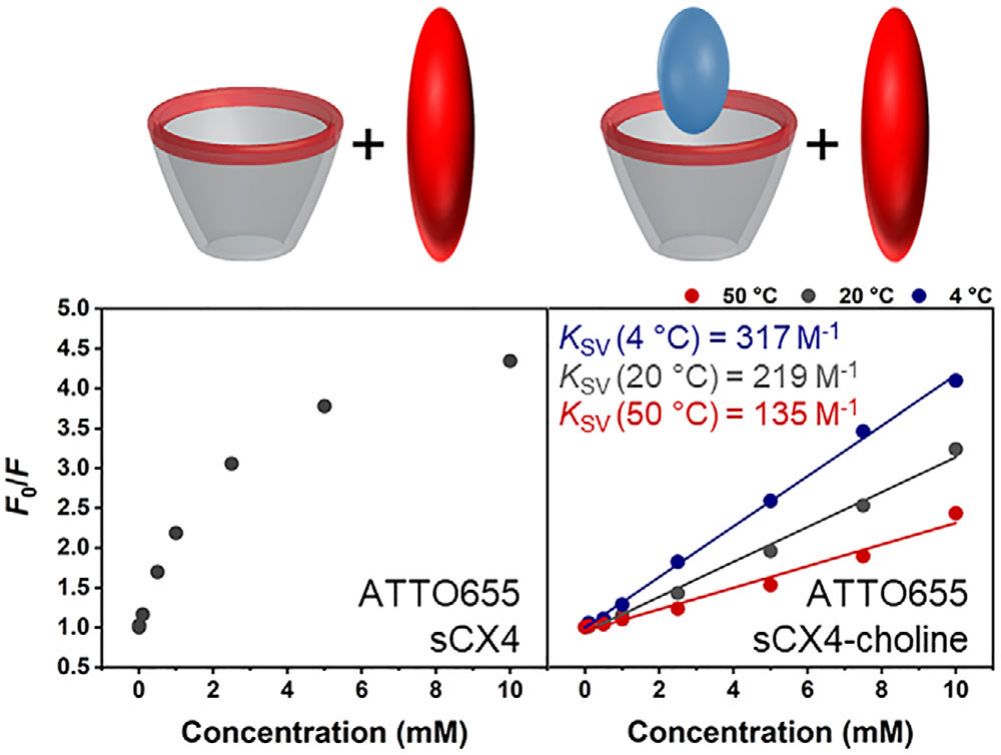
Stern–Volmer plots of ATTO655‐COOH with sCX4 in the absence (left) and presence of 12 mM choline (right). *F*_0_/*F* represents the ratio of fluorescence intensity in the absence (*F*_0_) to that in the presence (*F*) of the macrocyclic host. It is important to note that addition of 12 mM choline without sCX4 does not affect the fluorescence of ATTO655 (Figure S8).

Conversely, our data is compatible with a loose association of ATTO655 in an exclusion complex. To narrow down the possible interaction sites, i.e., the upper portal, the phenyl‐rings or the phenolic hydroxy groups at the lower portal, we used choline as a competitive guest. Choline is known to bind sCX4 at the upper portal and cavity stabilized by electrostatic interactions with the sulfonate acid groups and cation‐π interactions with the phenyl groups, respectively. The binding of choline was verified by an absorbance titration (Figure S9) and by ITC showing a characteristic salt dependence and high affinity (Figure S10). Strikingly, binding of choline to sCX4 does not prohibit simultaneous binding of ATTO655, but it alters the Stern–Volmer plot of ATTO655 to become nearly linear with a slight upward curvature (Figure 4). Under these conditions, ATTO655 remains bound, albeit with slightly reduced affinity (Figure S11a,b). This ternary complex shows the characteristic temperature (Figure 4) and lifetime dependence (Figure S11c,d) of static quenching and only minor contributions of dynamic quenching at higher concentrations of sCX4 >1 mM (Figures 4, S13). Furthermore, the interaction between ATTO655 and sCX4 has no dependence on the ionic strength of the buffer solution (Figure S12) in contrast to the interaction of sCX4 and choline (Figure S10). This finding is also different for LCG‐sCX4 [18, 47], in which increasing potassium concentrations displace LCG from the upper part of the cavity, leading to a 100‐fold decrease of the apparent binding affinity. The simultaneous binding of choline to the sCX4 cavity of the ATTO655‐sCX4 complex and the concomitant ∼10‐fold reduction of the ATTO655 binding affinity (Figure S11) present a case of allosteric regulation of the ATTO655 binding site reminiscent of allosteric regulation of conformational states or functions in proteins. We note that this analogy may be seen by some readers as site‐coupled ternary binding rather than allostery in a stricter biochemical sense. A possible interpretation of this finding is that ATTO655 binding to free sCX4 can occur to different conformations of the macrocycle [52], while choline stabilizes a subset of them.

**FIGURE 5:**
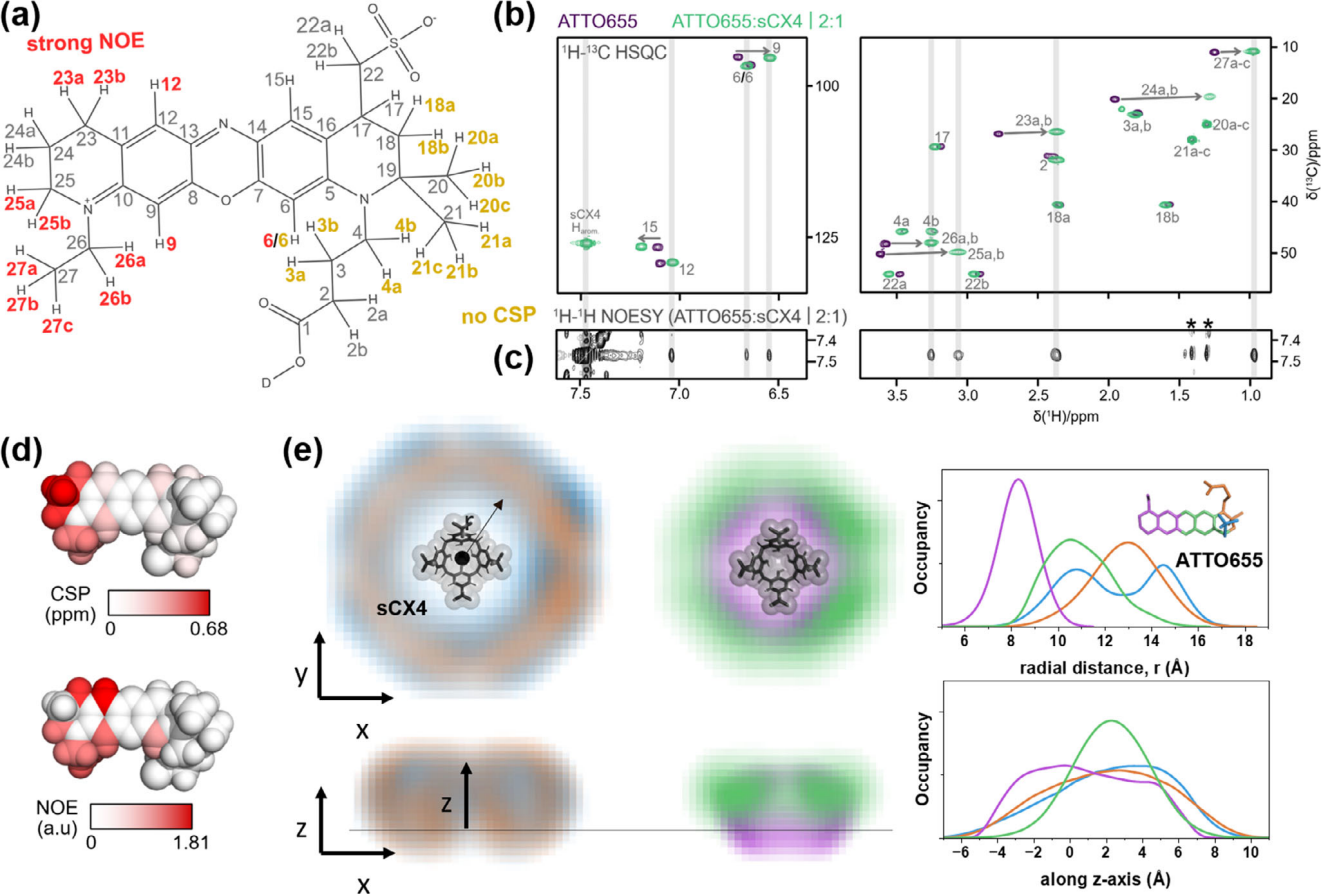
NMR measurements and structural modelling of ATTO655 and sCX4. (a) Consolidated information of spatial proximity (red) or detachment (yellow) of ATTO655 atoms from sCX4, as extracted from NMR. (b) Two‐dimensional ^1^H‐^13^C HSQC spectra in the absence (dark purple) and presence (green) of sCX4. Atoms where NMR signals remain at the same position after sCX4 addition are assumed to be remote from the interaction site. (c) NOESY spectrum recorded on the ATOO655:sCX4 mixture. Off‐diagonal peaks arise from cross‐relaxation and indicate spatial proximity. ^1^H chemical shifts of ATTO655 atoms that are spatially close to the aromatic ^1^H atom of sCX4 are highlighted with a gray stripe. Asterisks indicate artifact lines in the NOESY spectrum (d) CSP magnitudes (top) and NOESY cross peak intensities (bottom) visualized on ATTO655 with atoms shown as spheres. (e) Structural modelling of the ATTO655‐sCX4 complex. The model describes only the possible and sterically allowed locations of the ATTO655 dye. Further details on NMR data for structural assignment of ATTO655 and sCX4 and the derived model is provided in the Supporting Information.

As a consequence, binding interactions between ATTO655 and sCX4 have to occur in regions where ion–ion interactions are less relevant, e.g., at the lower phenolic portal or externally to the phenyl‐rings. To better understand the concrete geometry of binding, we performed NMR measurements that allow for deduction of spatial proximity (Figure 5). To extract information from NMR spectra, the observed signals need to be assigned to their corresponding ^1^H nucleus. This was achieved by combining information from the NOAH‐4 (BSCN) experiments [53] together with integrals and coupling patterns observed in the 1D ^1^H spectra. We analyzed ATTO655 (Figure 5a) and sCX4 in aqueous media both alone and in combination at different mixing ratios (Supporting Information). While ATTO655 yields detectable peaks for all protons, sensible information for sCX4 came only from the aromatic ^1^H nuclei. Signals from the methylene proteins (CH_2_ group) are strongly broadened, likely due to conformational dynamics in the NMR‐unfavorable µs‐regime.

We used two complementary kinds of NMR observables to extract information on proximity of ATTO655 moieties to the aromatic protons of sCX4. On one hand, we interrogated changes in the local magnetic field of each fluorophore proton (chemical‐shift perturbations, CSP, shown in Figure 5b) upon complex formation. Such CSPs are often used to assess interaction surfaces, e.g., in protein–ligand complexes [54]. The moiety‐specific extent of the CSP is represented in Figure 5d (top) on a space‐filling structure of ATTO655. We render it unlikely that the interaction of ATTO655 and sCX4 results in a complex with one unique binding geometry. In addition, the extent of chemical shift changes is not directly proportional to proximity in three‐dimensional space, especially when aromatic compounds with ring current effects are involved [54]. Therefore, we treat the magnitude of CSPs as a simple exclusion criterion: If the chemical shifts of ^1^H atoms of ATTO655 remain steady after addition of sCX4 (Δδ(^1^H) < 0.03 ppm), we conclude that those atoms do not participate in the interaction. Based on the comparison between two‐dimensional ^1^H‐^13^C HSQC correlations of either ATTO655 alone or an ATTO655:sCX4 (2:1) mixture (Figure 5b), it is evident that carbon atoms 3a‐b, 4a‐b, 18a‐b, 20a‐c, and 21a‐c of ATTO655 remain unperturbed (yellow labels in Figure 5a). All remaining atoms show significant chemical‐shift perturbations, except for atom 6, which exhibits only subtle changes. Based on this, we conclude that only one side of ATTO655, i.e., the one with the ethyl substituent interacts with sCX4, while the side with carboxy‐ and sulfonic‐acid substituents does not.

To complement those findings, we used two‐dimensional nuclear Overhauser effect spectroscopy (NOESY, Figure 5c), assessing through‐space magnetization transfer between calixarene and fluorophore. In this type of experiment, visible off‐diagonal signals (cross‐peaks) arise from nuclear spin cross‐relaxation, which occurs efficiently only up to a distance of ∼5‐6 Å. The aromatic ^1^H signal of sCX4, located at 7.48 ppm, shows intermolecular cross‐peaks to the atoms 6, 9, 12, 23a‐b, 25a‐b, 26a‐b, and 27a‐b of ATTO655^1^, which is again depicted in the form of a color gradient on the ATTO655 structure in Figure 5d (bottom). Adding these confirmed proximities into the consolidated representation shown in Figure 5a (red labels) shows that both types of data, CSPs and NOESY cross peak intensities, are in full agreement with each other.

Based on the obtained experimental restrains from NMR, we modelled a spatial ensemble of the ATTO655‐sCX4 complex (Figure 5e) consistent with the data using the integrative modelling platform [55]. The NOESY‐restraints were modeled as asymmetric flat‐bottom harmonic upper‐bound potentials evaluated against all four equivalent proton pairs with only the closest pair contributing to the score. CSP restraints were modeled as lower‐bound potentials applied to all four equivalent pairs simultaneously (details in Supporting Information). The resulting structural ensemble was analyzed by projecting atomic positions onto a three‐dimensional voxel grid to yield insertion depth profiles, lateral radial distributions, and dye‐plane orientation histograms, highlighting configurations of the ATTO655‐sCX4 complex that are compatible with the data (Figure 5e). The spatial ensemble shows no inclusion of the dye into the interior of the cavity or horizontal binding to its phenolic rim. Instead, the integrative model suggests the formation of a complex in which the guest binds to the outer surface of the host (exclusion complex). Preferred orientations of ATTO655 relative to sCX4 are revealed by the marginal radial density distributions (Figure 5e). Specifically, the magenta density indicates ATTO655 binding distal to the sulfonate groups. In contrast, the orange linker‐region density suggests that longer linkers of ATTO655 with matching positive charges could enable additional, higher‐affinity binding through the classical sCX4 interface, which is also used in the case of choline binding.

### 2.4 Single‐molecule Spectroscopy of Macrocycle‐dye Complexes

**FIGURE 6:**
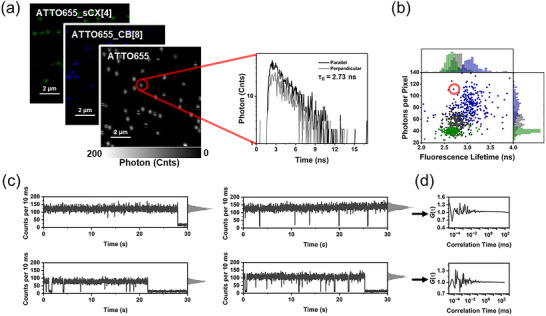
(a) Representative single‐molecule FLIM images of immobilized ATTO655‐dsDNA and in the absence (black) and presence of 25 µM CB8 (blue) or 10 mM sCX4 (green). The red circle indicates the TCSPC histogram of a selected dye molecule. (b) 2D histogram plotting fluorescence lifetime values of individual dyes versus photon counts per pixel, providing quantitative insights into photophysical changes. Representative fluorescence intensity traces of immobilized ATTO655‐dsDNA and (d) corresponding autocorrelation curves.

To understand further details in the photophysical interplay of ATTO655 and the macrocycles, but also to pave the way for applications of similar complexes in biophysical and biochemical assays [56], single‐molecule research and super‐resolution microscopy [31], we investigated their interaction on the single‐dye level. Our rationale was to assess static and dynamic heterogeneity in the complex properties [5, 20, 26] and to disentangle association and dissociation from other photophysical effects. Since relevant applications of dye molecules in biosensing or biochemistry require their coupling to biomacromolecules, we used a 20‐mer oligonucleotide structure with terminal 5'‐attachment of ATTO655 [42], in which the dye is protected from interactions with the glass surfaces. Surface‐immobilization of the ATTO655‐dsDNA construct was achieved via biotin‐streptavidin interactions on PEGylated glass coverslips (Figure 6a; see also Figure S16). The immobilization approach eliminated issues related to aggregation of dye‐labelled dsDNA in solution upon addition of the macrocycles.^2^

By diluting the sample to picomolar concentrations, we achieved a molecular density that allowed for the observation of individual fluorescent spots via confocal scanning microscopy. Analysis of FLIM images of isolated fluorescence spots in multiple regions of interest (ROI) showed that immobilized ATTO655‐dsDNA exhibited a mean fluorescence lifetime of 2.77 ns (Figure 6a), which is approximately 0.8 ns longer than that of free ATTO655 in solution (Figures 2, 3). This increase is attributed to a shielding effect of the DNA molecule, which reduces the water accessibility of the dye, mitigating water‐induced quenching [1] or allows interactions of ATTO655 with the DNA, e.g., (end‐)stacking or groove‐binding. Upon addition of 25 µM CB8, the fluorescence lifetime increased to 3.05 ns, accompanied by a two‐fold enhancement in brightness (Figure 6b). Meanwhile, the addition of 10 mM sCX4 reduced the fluorescence intensity by 30% and the lifetime to an average value of 2.65 ns. It was clearly visible that the spread of lifetime values for ATTO655‐CB8 was much larger than for ATTO655 on dsDNA alone, indicating that the formed complexes introduce an additional layer of variations in the microenvironment of the dye. Notably, the lifetime distribution for ATTO655‐sCX4 became quite narrow and well‐defined (Figure 6b).

The general trends in both lifetime and intensity were consistent with the behavior of ATTO655 observed in solution (Figures 2, 3). The reduced magnitude of the effects in comparison to the solution data, however, indicates a lower affinity of the macrocycles for the dye on the dsDNA as also seen in the Stern–Volmer plot (Figure S15). This could be rationalized by the strong negative charge of the dsDNA backbone or a reduced accessibility of the dye. Unfortunately, higher concentrations of both macrocycles could not be applied in our experiments due to the limited solubility of CB8 and acidification of the low‐salt buffer solution by sCX4 at concentrations >10 mM.

We next used the unique capability of confocal scanning microscopy to monitor the fluorescence intensity and lifetime of individual molecules over extended time periods with high temporal resolution [57, 58]. Representative fluorescence trajectories of ATTO655‐dsDNA excited with 5 µW laser power (1.7 kW cm^−2^) (Figure 6c) displayed continuous fluorescence emission with a fluorescence lifetime of the on‐state of 2.79 ± 0.06 ns. We observed no blinking on timescales from 10 ms to 100 ns seen by a missing bunching amplitude in the autocorrelation analysis (Figure 6d). Occasional slow off‐blinks were visible in the traces together with irreversible photobleaching on the timescale of tens of seconds (Figure 6c), which is consistent with previous work [42]. Overall, ATTO655 showed homogeneous photophysical behavior on dsDNA, well represented by the traces in Figure 6c (see more traces in Figure S17).

**FIGURE 7:**
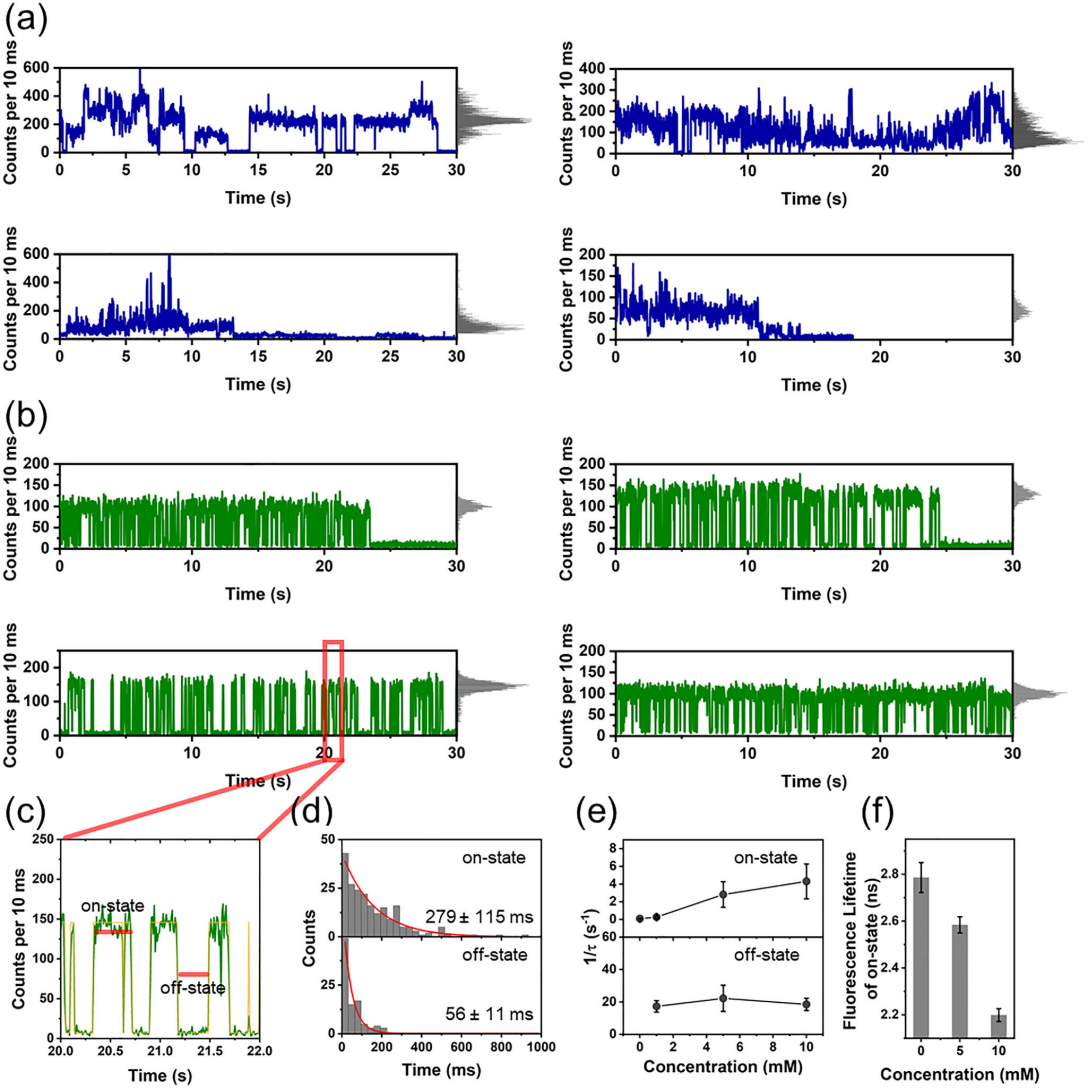
Single‐molecule FLIM and kinetic analysis of ATTO655‐dsDNA interactions with macrocyclic hosts. (a, b) Representative fluorescence traces of immobilized ATTO655‐dsDNA in the presence of (a) 25 µM CB8 and (b) 10 mM sCX4. (c) Zoomed‐in trace segment overlaid with a two‐state hidden Markov model fit (yellow line). (d) Dwell time histograms for the on‐ and off‐states with mono‐exponential fits (red lines). (e) Plot of the inverse on‐ and off‐state dwell time (1/*τ*) versus sCX4 concentration, demonstrating the concentration dependence of the blinking kinetics. (f) Fluorescence lifetime of the on‐state as a function of sCX4 concentration.

To better understand how macrocycle binding affects the fluorescence of individual ATTO655 molecules, we first approximated the dissociation rate constants (*k*_off_) from stopped‐flow experiments with fluorescence detection (Supporting Information) in combination with our FCS data (Figures 2, 3). We found the dissociation rate constant *k*_off_ to be on the order of ∼50 s^−1^ and ∼10^5^ s^−1^ for ATTO655‐CB8 and ATTO655‐sCX4, respectively. These values are in good agreement with previous data on pyronine and cyclodextrins or complexes of CB7 with similar guests [5, 59]. Importantly, these values give an upper barrier for the residence time of the macrocycles on the dye, i.e., the timescale in the traces where we expect altered photophysical properties compared to ATTO655 (see Figures 6, 7).

ATTO655‐dsDNA exhibited a large variability in its brightness in the presence of CB8 and showed frequent blinking on a range of timescales (Figures 7a, S18). Such irregular intensity changes reflect a combination of complex dissociation and association on the millisecond timescale, multiple binding modalities of dye to the macrocycle and photophysical blinking, e.g., when the dye is transiently bound inside the nonpolar cavity and less exposed to water and molecular oxygen. Although a relation between the intensity of the fluorescence signal and the fluorescence lifetime could be established (Figure S19), the nonuniform signals offer limited information on the concrete mechanism of complex formation and dissociation.

The obtained traces of ATTO655‐dsDNA with sCX4 on the other hand were quite homogeneous and showed blinking events with millisecond durations with a frequency that correlated with the sCX4 concentration (Figures 7b, S20). Through the analysis by a two‐state hidden Markov model (Figure 7c,d), the dwells of the on/off states were quantified in a concentration‐dependent manner. Exponential fitting of the on‐ (*τ*_on_) and off‐state (*τ*_off_) dwell‐time histograms show that only *τ*_on_ becomes shorter, while *τ*_off_ remains constant for increasing concentrations of sCX4 (Figure 7e, 0–10 mM). The observed off‐dwells with a duration of *τ*_off_(sCX4) ∼50 ms showed only background signal, i.e., scattering (Figure S21) and thus represent a true off‐state. Furthermore, excitation‐intensity dependent experiments showed that this off‐state is caused by a photoinduced process, in which the frequency of events increases with laser intensity (Figure S22). State‐dependent fluorescence lifetime analysis (Figure 7f) also showed a concentration‐dependent lifetime decay of the on‐state with shorter lifetimes at higher sCX4 concentrations.

**FIGURE 8:**
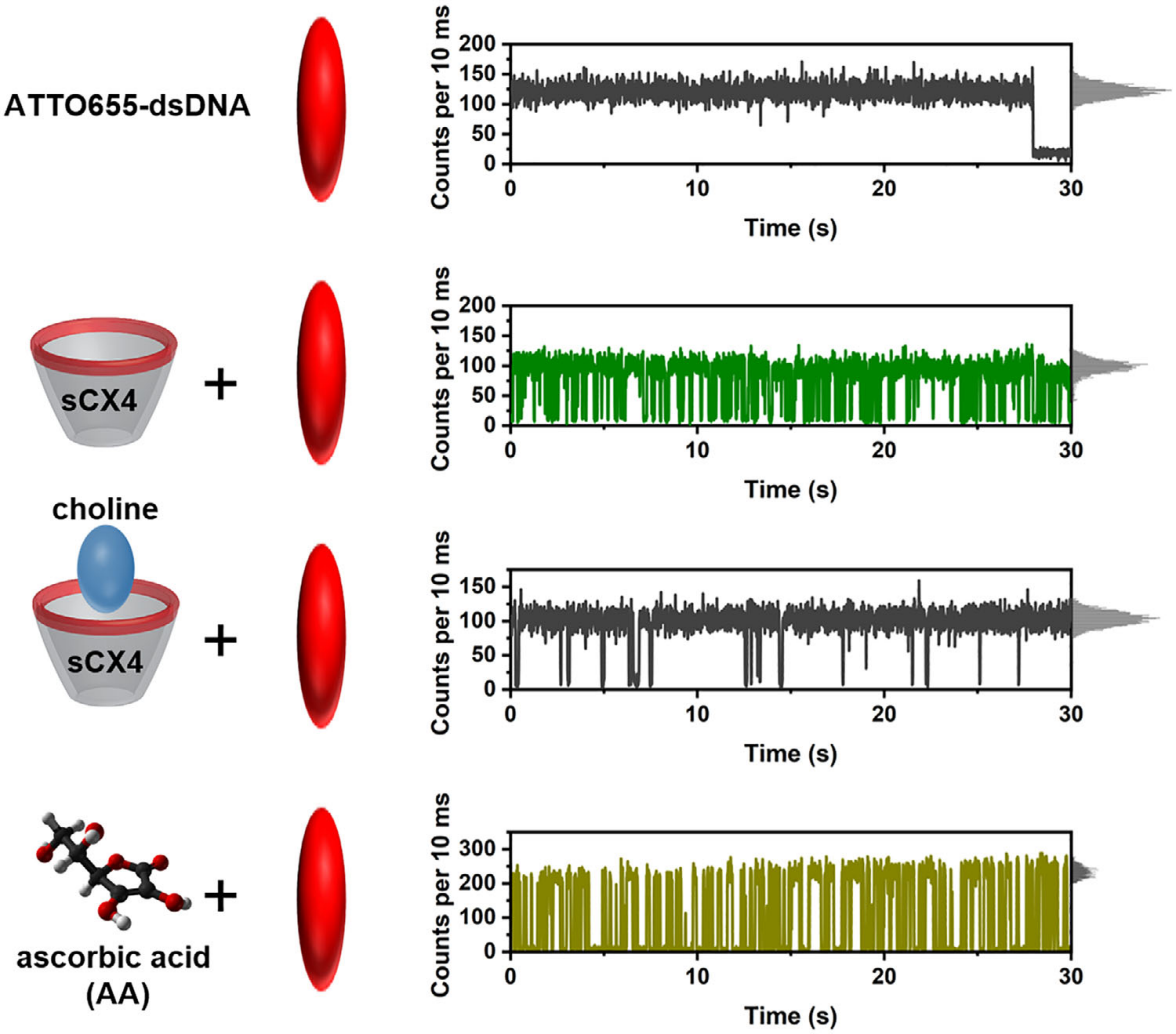
Single‐molecule fluorescence traces of ATTO655‐dsDNA. Conditions (top to bottom): buffer control, 10 mM sCX4, 10 mM sCX4 with 11 mM choline, and 10 µM AA. See Figures S17, S20, S23, and S25 for extended data.

Our interpretation of the above data is that the on‐states in Figure 7b represent the fast (time‐averaged) µs‐equilibrium between free and sCX4‐bound ATTO655 [59, 60]. The observed reduction of fluorescence lifetime and intensity in the on‐state with higher sCX4 concentrations are consequently caused by combined static and dynamic quenching by sCX4. The slow blinking events in the single‐molecule traces on the other hand are caused by a photoinduced process due to its excitation intensity dependence (Figure S22). One possible explanation is that photoinduced electron transfer (PET) converts ATTO655 into a nonfluorescent radical ion, which is reactivated by oxidation via molecular oxygen (τ_off_(sCX4) ∼50 ms). This photochemical reaction pathway can be induced by collision or binding of ATTO655 either in its singlet‐ or triplet state to the sCX4 host. To support this PET‐hypothesis, we compared the behavior of sCX4 with that of ascorbic acid (AA). This strong redox agent (*E*_ox_ = 0.06 V, SCE) is known to generate reduced ATTO655 species with very similar blinking signatures [42]. AA induced dynamic quenching seen by a Stern–Volmer analysis (Figure S24) with notable singlet quenching >1% at AA concentrations > 1 mM. However, already 2–10 µM of AA were sufficient to induce frequent blinking events with a dwell time of *τ*_off_(AA) ∼170 ms in single‐molecule traces, i.e., are substantially longer than for sCX4 (Figures 7, 8, S25/S26). The resulting off‐states were previously attributed to the formation of a radical anion for AA, from which the singlet state can be recovered by oxygen [42]. The Rehm–Weller equation^3^ allows to calculate the thermodynamic driving forces for PET [61]. ATTO655 can be reduced to a non‐fluorescent radical anion or its protonated leuco‐form via one‐ or two‐electron reactions (*E*_red_ = −0.42 V vs. SCE (radical anion), *E*_red_ = −0.7 V vs. SCE (leuco form)). As suggested previously [42], reduction of ATTO655 mainly originates from the reaction of AA with the ATTO655 triplet T_1_, since it has a much longer lifetime than the singlet state S_1_. PET would be generally exergonic for AA according to the Rehm–Weller equation. For sCX4 (*E*_ox_ ∼ 0.82 V, SCE), however, only the one‐electron reduction of ATTO655 to the radical anion has negative Δ*G* ^0^‐values. Thus, the off‐state dwell‐time differences between *τ*_off_(sCX4) ∼50 ms and *τ*_off_(AA) ∼170 ms might be a result of the different oxidation states of ATTO655 or caused by completely distinct photochemical reactions.

The off‐blinking in the single‐molecule traces is strongly excitation‐intensity dependent, yet the Stern–Volmer plots of free ATTO655 in the fluorimeter do not change notably for different excitation conditions (Figure S27). Although this might not be surprising considering that the excitation densities are vastly different between single‐molecule and bulk experiments, it would be interesting to understand whether off‐blinking in ATTO655‐sCX4 is related to complex formation or mere collisions between the ATTO655 triplet (as for AA; Figure 8) and sCX4. Also, the fact that the interaction strength of ATTO655 with sCX4 is reduced when bound to dsDNA suggests that additional factors such as the dye microenvironment or distinct interactions of different sCX4 conformational states with the dye influence the photophysics.

Ultimately, we wondered whether the host‐induced photochemical reaction pathway could be modulated by additional binding of choline to the ATTO655‐sCX4 complex. Our results showed that the blinking frequency associated with the photochemical conversion into a dark state is strongly reduced when choline occupies the sCX4 cavity (Figure 8, Figure S23). Again, we observe an allosteric modulation this time of a chemical reaction reminiscent of enzymatic reactions, where binding of a noncompetitive inhibitor to a remote site decreases the reaction rate at the catalytic reaction center.

In summary, our data shows that the upper portal of sCX4 with its sulfonic acid moieties is responsible for photo‐induced off‐switching in single‐molecule trajectories of ATTO655. Currently, PET remains a possible mechanistic explanation for this, yet we are unable to take a firm decision on this since additional support on the appearance of radicals, e.g., via EPR, would be necessary.

## 3 Conclusion

Herein, we investigated host–guest complexation of the oxazine dye ATTO655 with the supramolecular hosts CB8 and sCX4 (Figure 1). Although complexation with CB8 proceeds as expected forming an inclusion complex with an enhanced brightness (Figure 1b), an inclusion into the CX4 hydrophobic cavity and concomitant fluorescence quenching is not observed. Instead, our study revealed an unexpected external binding of ATTO655 around the aromatic moieties of sCX4 leading to the formation of quenched dim complexes. Consequently, the sulfonate‐groups and hydrophobic cavity of CX4 remain accessible for known guests of sCX4 such as the neurotransmitter choline leading to the formation of a ternary complex. In this complex, choline binding reduces the binding affinity of ATTO655 and prevents the formation of a photoinduced dark state observed in single‐molecule traces, which presents a form of allosteric regulation of binding and the photoinduced processes. Although our work reveals various unprecedented mechanistic features of macrocycle‐dye complexes, we believe that the technical advances introduced, namely the use of single molecule microscopy as well as a quantitative integrative modelling approach with explicit treatment of experimental uncertainty not used so far [62, 63], will be of use for the supramolecular chemistry community.

Besides the detailed insight into photophysical mechanisms, the present work is also fundamental for future applications of similar complexes. Since ATTO655 and similar dyes [42, 64, 65] are excellent candidates for applications in supramolecular assays, e.g., indicator displacement and tandem assays, an essential point would be to increase the affinity between dye and host and to further optimize the quenching. This could be done by synthetic modifications of dyes and hosts. The development of such applications will also benefit from further structural elucidation of the complexes and a better understanding of the quenching mechanisms involved. For example, the nature of the dark‐state seen in the single‐molecule traces remains at least to some degree elusive and its relation to quenching of the free and dsDNA‐bound ATTO655 is currently unclear.

Overall, we believe that this study outlines the great potential of single‐molecule spectroscopy for detailed photophysical investigations of dye‐macrocycle complexes, which will enable serendipitous discoveries and provide new fundamental insights for the rationale design of assays based on the interaction of dyes and macrocycles required for advanced bioimaging and sensing applications.

## Author Contributions

Thorben Cordes conceived and designed the study. Thomas Peulen, Rasmus Linser, and Thorben Cordes supervised the project. Rasmus Linser and Thorben Cordes acquired funding. Siyu Lu, Thomas Peulen, Hongbin Wu, Florian Lindemann performed research. Siyu Lu, Thomas Peulen, Hongbin Wu, Florian Lindemann and Thorben Cordes analyzed data. Thomas Peulen provided new software for data analysis and integrative modelling. Siyu Lu and Thorben Cordes wrote the initial draft of the paper. All authors contributed to the discussion and interpretation of the results and edited the final version of the paper.

## Materials and methods

A detailed description of materials and methods used in this paper are provided in the Supporting Information (Supplementary Note 3).

## Conflicts of Interest

T.C. is a scientific cofounder and share‐holder of FluoBrick Solutions GmbH, a company that distributes fluorescence microscopy and spectroscopy instruments.

## Supporting information




**Supporting File 1**: anie72911‐sup‐0001‐SuppMat.docx.

## Data Availability

The data that support the findings of this study are available from the corresponding author upon reasonable request.

## ENDNOTES

1 The cross‐peak to atom 6 is fairly weak and likely more remote than the others, in agreement with its small CSP described above. Although a cross‐peak to atoms 24a‐b is expected from the structure of ATTO665, this cannot be unambiguously determined from the shown data alone. Its peak position at 1.3 ppm overlaps with 20a‐c, and the artifact lines caused by this methyl group in the NOESY spectrum mask any other signal (indicated by an asterisk). However, our full dataset indicates that 1H atoms 24a‐b do indeed interact with sCX4 (Supporting Information).

2 Analysis of diffusing ATTO655‐dsDNA showed aggregation, i.e., a strongly diminished burst frequency and the appearance of large and long‐lasting signals, upon addition of both macrocycles. The latter were way above the expected count rates and burst durations of individual dye molecules, which typically do not exceed 0.1–1 MHz (see FCS data in Figure 2/3 or single‐molecule traces in Figure S14).

3 Δ*G* ^0^ = e[*E*_ox_ – *E*_red_] – *E*_0,0_ + C with the oxidation (*E*_ox_) and reduction potential (*E*_red_) of donor (oxidant) and acceptor (reductant) and zero‐zero energy (E_0,0_). We used published values for ATTO655: E_0,0_ = 1.86 eV (S_1_), E_0,0_ = 1.56 eV (T_1_), E_red_ = −0.42 V versus SCE (radical anion), *E*_red_ = ‐0.7 V versus SCE (leuco form) and assume the constant C to be zero due to the polar solvent water [46].

## References

[1] L. Briant , J. Maillard , and A. Fürstenberg , “Super‐resolution Imaging With a Cucurbituril‐encapsulated Fluorophore,” Chemical Communications 60 (2024): 13943–13946, 10.1039/D4CC05274A. 39574385 PMC11563205

[2] M. G. Rasmussen , G. Masciotta , K. H. Hansen , et al., “Fluorescent Probe for δ‐Cyclodextrin Enables Guest Encapsulation Studies Via an Indicator Displacement Assay,” ChemistryEurope 3 (2025): e202500029, 10.1002/ceur.202500029.

[3] D. Shetty , J. K. Khedkar , K. M. Park , and K. Kim , “Can We Beat the Biotin–avidin Pair?: Cucurbit[7]Uril‐based Ultrahigh Affinity Host–guest Complexes and Their Applications,” Chemical Society Reviews 44 (2015): 8747–8761, 10.1039/C5CS00631G. 26434388

[4] M. Sayed , D. K. Maity , and H. Pal , “Cucurbit[7/8]Uril Assisted Modulations in the Photophysical Properties of 4′,6‐diamidino‐2‐phenylindole Dye,” Journal of Photochemistry and Photobiology A: Chemistry 445 (2023): 115088, 10.1016/j.jphotochem.2023.115088.

[5] W. Al‐Soufi , B. Reija , M. Novo , S. Felekyan , R. Kühnemuth , and C. A. M. Seidel , “Fluorescence Correlation Spectroscopy, a Tool to Investigate Supramolecular Dynamics: Inclusion Complexes of Pyronines with Cyclodextrin,” Journal of the American Chemical Society 127 (2005): 8775–8784, 10.1021/ja0508976. 15954784

[6] H. Bakirci , A. L. Koner , T. Schwarzlose , and W. M. Nau , “Analysis of Host‐Assisted Guest Protonation Exemplified for p ‐Sulfonatocalix[4]Arene—Towards Enzyme‐Mimetic p Ka Shifts,” Chemistry A European Journal 12 (2006): 4799–4807, 10.1002/chem.200501479. 16673426

[7] R. Sasmal , A. Som , P. Kumari , et al., “Supramolecular Guest Exchange in Cucurbit[7]Uril for Bioorthogonal Fluorogenic Imaging across the Visible Spectrum,” ACS Central Science 10 (2024): 1945–1959, 10.1021/acscentsci.4c01080. 39463826 PMC11503495

[8] D. Kim , A. Aktalay , N. Jensen , et al., “Supramolecular Complex of Photochromic Diarylethene and Cucurbit[7]Uril: Fluorescent Photoswitching System for Biolabeling and Imaging,” Journal of the American Chemical Society 144 (2022): 14235–14247, 10.1021/jacs.2c05036. 35895999 PMC9376957

[9] I. Roy , S. Bobbala , J. Zhou , et al., “ExTzBox: A Glowing Cyclophane for Live‐Cell Imaging,” Journal of the American Chemical Society 140 (2018): 7206–7212, 10.1021/jacs.8b03066. 29771509

[10] Y.‐C. Pan , J.‐H. Tian , and D.‐S. Guo , “Molecular Recognition with Macrocyclic Receptors for Application in Precision Medicine,” Accounts of Chemical Research 56 (2023): 3626–3639, 10.1021/acs.accounts.3c00585. 38059474

[11] I. Roy , S. Bobbala , R. M. Young , et al., “A Supramolecular Approach for Modulated Photoprotection, Lysosomal Delivery, and Photodynamic Activity of a Photosensitizer,” Journal of the American Chemical Society 141 (2019): 12296–12304, 10.1021/jacs.9b03990. 31256588

[12] L. Zhang , C. Wang , Y. Li , et al., “Modular Design and Scaffold‐Synthesis of Multi‐Functional Fluorophores for Targeted Cellular Imaging and Pyroptosis,” Angewandte Chemie International Edition 64 (2025): e202415627, 10.1002/anie.202415627. 39555698 PMC11753610

[13] I. Roy , A. Garci , Y. Beldjoudi , et al., “Host–Guest Complexation‐Mediated Supramolecular Photon Upconversion,” Journal of the American Chemical Society 142 (2020): 16600–16609, 10.1021/jacs.0c05445. 32865399

[14] A. Saifi , J. P. Joseph , A. P. Singh , A. Pal , and K. Kumar , “Complexation of an Azo Dye by Cyclodextrins: A Potential Strategy for Water Purification,” ACS Omega 6 (2021): 4776–4782, 10.1021/acsomega.0c05684. 33644585 PMC7905815

[15] N. Dash , F. A. S. Chipem , and G. Krishnamoorthy , “Encapsulation of 2‐(4′‐N,N‐dimethylamino)phenylimidazo[4,5‐b]Pyridine in β‐cyclodextrin: Effect on H‐bond‐induced Intramolecular Charge Transfer Emission,” Photochemical & Photobiological Sciences 8 (2009): 1708–1715, 10.1039/b9pp00023b. 20024168

[16] S. V. Patil , S. V. Athare , A. Jagtap , K. M. Kodam , S. P. Gejji , and D. D. Malkhede , “Encapsulation of Rhodamine‐6G Within P‐Sulfonatocalix[n]arenes: NMR, Photophysical Behaviour and Biological Activities,” RSC Advances 6 (2016): 110206–110220, 10.1039/C6RA23614F.

[17] X. Y. Hu , Y. Y. Wang , H. B. Li , and D. S. Guo , “Fluorescence Enhancement by Calixarene Supramolecular Aggregate,” Molecules 25 (2020): 5912, 10.3390/molecules25245912. 33327371 PMC7764848

[18] N. Lavande , A. Acuna , N. Basilio , V. Francisco , D. D. Malkhede , and L. Garcia‐Rio , “A Journey from calix[4]arene to calix[6] and calix[8]arene Reveals More than a Matter of Size. Receptor Concentration Affects the Stability and Stoichiometric Nature of the Complexes,” Physical Chemistry Chemical Physics 19 (2017): 13640–13649, 10.1039/C7CP01889D. 28530732

[19] M. Sayed , M. Sundararajan , J. Mohanty , A. C. Bhasikuttan , and H. Pal , “Photophysical and Quantum Chemical Studies on the Interactions of Oxazine‐1 Dye with Cucurbituril Macrocycles,” Journal of Physical Chemistry B 119 (2015): 3046–3057, 10.1021/jp509243j. 25601388

[20] T. A. Martyn , J. L. Moore , R. L. Halterman , and W. T. Yip , “Cucurbit[7]Uril Induces Superior Probe Performance for Single‐Molecule Detection,” Journal of the American Chemical Society 129 (2007): 10338–10339, 10.1021/ja073996n. 17676851

[21] W. M. Nau and J. Mohanty , “Taming Fluorescent Dyes With Cucurbituril,” International Journal of Photoenergy 7 (2005): 133–141, 10.1155/S1110662X05000206.

[22] R. N. Dsouza , U. Pischel , and W. M. Nau , “Fluorescent Dyes and Their Supramolecular Host/Guest Complexes With Macrocycles in Aqueous Solution,” Chemical Reviews 111 (2011): 7941–7980, 10.1021/cr200213s. 21981343

[23] M. A. Alnajjar , W. M. Nau , and A. Hennig , “A Reference Scale of Cucurbit[7]Uril Binding Affinities,” Organic & Biomolecular Chemistry 19 (2021): 8521–8529, 10.1039/D1OB01304A. 34378628

[24] S. J. Barrow , S. Kasera , M. J. Rowland , J. del Barrio , and O. A. Scherman , “Cucurbituril‐Based Molecular Recognition,” Chemical Reviews 115 (2015): 12320–12406, 10.1021/acs.chemrev.5b00341. 26566008

[25] K. I. Assaf and W. M. Nau , “Cucurbiturils: From Synthesis to High‐affinity Binding and Catalysis,” Chemical Society Reviews 44 (2015): 394–418, 10.1039/C4CS00273C. 25317670

[26] A. McLean , R. L. Sala , B. W. Longbottom , et al., “Single‐Molecule Stoichiometry of Supramolecular Complexes,” Journal of the American Chemical Society 146 (2024): 12877–12882, 10.1021/jacs.4c00611. 38710014 PMC11100007

[27] A. L. Koner and W. M. Nau , “Cucurbituril Encapsulation of Fluorescent Dyes,” Supramolecular Chemistry 19 (2007): 55–66, 10.1080/10610270600910749.

[28] P. Montes‐Navajas , A. Corma , and H. Garcia , “Complexation and Fluorescence of Tricyclic Basic Dyes Encapsulated in Cucurbiturils,” Chemphyschem 9 (2008): 713–720, 10.1002/cphc.200700735. 18330855

[29] F. Biedermann , E. Elmalem , I. Ghosh , W. M. Nau , and O. A. Scherman , “Strongly Fluorescent, Switchable Perylene Bis(diimide) Host–Guest Complexes with Cucurbit[8]Uril in Water,” Angewandte Chemie International Edition 51 (2012): 7739–7743, 10.1002/anie.201202385. 22730071

[30] L. Briant and A. Fürstenberg , “Enhancing the Brightness of Red‐emitting Fluorophores in Aqueous Solution by Molecular Encapsulation,” Chimia 79 (2025): 259–262, 10.2533/chimia.2025.259. 40314303

[31] D. Kolarski , M. L. Bossi , R. Lincoln , J. C. Fuentes‐Monteverde , V. N. Belov , and S. W. Hell , “Supramolecular Complexation of Quenched Rosamines with Cucurbit[7]Uril: Fluorescence Turn‐ON Effect for Super‐Resolution Imaging,” Journal of the American Chemical Society 147 (2025): 28893–28902, 10.1021/jacs.5c06406. 40731377 PMC12356589

[32] S. Doose , H. Neuweiler , and M. Sauer , “A Close Look at Fluorescence Quenching of Organic Dyes by Tryptophan,” Chemphyschem 6 (2005): 2277–2285, 10.1002/cphc.200500191. 16224752

[33] C. Eggeling , J. Widengren , L. Brand , J. Schaffer , S. Felekyan , and C. A. Seidel , “Analysis of Photobleaching in Single‐Molecule Multicolor Excitation and Förster Resonance Energy Transfer Measurements,” Journal of Physical Chemistry A 110 (2006): 2979–2995, 10.1021/jp054581w. 16509620

[34] S. Kuznetsova , G. Zauner , T. J. Aartsma , et al., “The Enzyme Mechanism of Nitrite Reductase Studied at Single‐molecule Level,” Proceedings National Academy of Science USA 105 (2008): 3250–3255, 10.1073/pnas.0707736105. PMC2265120 18303118

[35] N. Karedla , A. I. Chizhik , I. Gregor , A. M. Chizhik , O. Schulz , and J. Enderlein , “Single‐Molecule Metal‐Induced Energy Transfer (smMIET): Resolving Nanometer Distances at the Single‐Molecule Level,” Chemphyschem 15 (2014): 705–711, 10.1002/cphc.201300760. 24478241

[36] C. N. Hulleman , W. Li , I. Gregor , B. Rieger , and J. Enderlein , “Photon Yield Enhancement of Red Fluorophores at Cryogenic Temperatures,” Chemphyschem 19 (2018): 1774–1780, 10.1002/cphc.201800131. 29659104

[37] M. Heilemann , S. van de Linde , A. Mukherjee , and M. Sauer , “Super‐Resolution Imaging with Small Organic Fluorophores,” Angewandte Chemie International Edition 48 (2009): 6903–6908, 10.1002/anie.200902073. 19670280

[38] R. Wombacher , M. Heidbreder , S. van de Linde , et al., “Live‐cell Super‐resolution Imaging With Trimethoprim Conjugates,” Nature Methods 7 (2010): 717–719, 10.1038/nmeth.1489. 20693998

[39] S. van de Linde , M. Heilemann , and M. Sauer , “Live‐cell Super‐Resolution Imaging With Synthetic Fluorophores,” Annual Review of Physical Chemistry 63 (2012): 519–540, 10.1146/annurev-physchem-032811-112012. 22404589

[40] E. Ploetz , B. Visser , W. Slingenbergh , et al., “Selective Functionalization of Patterned Glass Surfaces,” Journal of Materials Chemistry B 2 (2014): 2606–2615, 10.1039/C3TB21763A. 32261427

[41] R. Jungmann , C. Steinhauer , M. Scheible , A. Kuzyk , P. Tinnefeld , and F. C. Simmel , “Single‐Molecule Kinetics and Super‐Resolution Microscopy by Fluorescence Imaging of Transient Binding on DNA Origami,” Nano Letters 10 (2010): 4756–4761, 10.1021/nl103427w. 20957983

[42] J. Vogelsang , T. Cordes , C. Forthmann , C. Steinhauer , and P. Tinnefeld , “Controlling the Fluorescence of Ordinary Oxazine Dyes for Single‐Molecule Switching and Superresolution Microscopy,” Proceedings National Academy of Science USA 106 (2009): 8107–8112, 10.1073/pnas.0811875106. PMC2688868 19433792

[43] Q. Zheng , S. Jockusch , Z. Zhou , et al., “Electronic Tuning of Self‐healing Fluorophores for Live‐cell and Single‐molecule Imaging,” Chemical Science 8 (2017): 755–762, 10.1039/C6SC02976K. 28377799 PMC5299821

[44] J. Kim , I.‐S. Jung , S.‐Y. Kim , et al., “New Cucurbituril Homologues: Syntheses, Isolation, Characterization, and X‐ray Crystal Structures of Cucurbit[ n ]uril ( n = 5, 7, and 8),” Journal of the American Chemical Society 122 (2000): 540–541, 10.1021/ja993376p.

[45] S. Shinkai , K. Araki , and O. Manabe , “Does the Calixarene Cavity Recognise the Size of Guest Molecules? On the ‘Hole‐size Selectivity’ in Water‐soluble Calixarenes,” Journal of the Chemical Society, Chemical Communications (1988): 187–189, 10.1039/C39880000187.

[46] T. Dertinger , V. Pacheco , I. von der Hocht , R. Hartmann , I. Gregor , and J. Enderlein , “Two‐focus Fluorescence Correlation Spectroscopy: A New Tool for Accurate and Absolute Diffusion Measurements,” Chemphyschem 8 (2007): 433–443, 10.1002/cphc.200600638. 17269116

[47] D.‐S. Guo , V. D. Uzunova , X. Su , Y. Liu , and W. M. Nau , “Operational Calixarene‐based Fluorescent Sensing Systems for Choline and Acetylcholine and Their Application to Enzymatic Reactions,” Chemical Science 2 (2011): 1722, 10.1039/C1SC00231G.

[48] M. R. Eftink and C. A. Ghiron , “Fluorescence Quenching Studies with Proteins,” Analytical Biochemistry 114 (1981): 199–227, 10.1016/0003-2697(81)90474-7. 7030122

[49] D. Genovese , M. Cingolani , E. Rampazzo , L. Prodi , and N. Zaccheroni , “Static Quenching Upon Adduct Formation: A Treatment without Shortcuts and Approximations,” Chemical Society Reviews 50 (2021): 8414–8427, 10.1039/D1CS00422K. 34142693

[50] J. Maillard , K. Klehs , C. Rumble , E. Vauthey , M. Heilemann , and A. Fürstenberg , “Universal Quenching of Common Fluorescent Probes by Water and Alcohols,” Chemical Science 12 (2021): 1352–1362, 10.1039/d0sc05431c. PMC8179231 34163898

[51] W. M. Nau , M. Florea , and K. I. Assaf , “Deep inside Cucurbiturils: Physical Properties and Volumes of Their Inner Cavity Determine the Hydrophobic Driving Force for Host–Guest Complexation,” Israel Journal of Chemistry 51 (2011): 559–577, 10.1002/ijch.201100044.

[52] A. Ikeda and S. Shinkai , “Novel Cavity Design Using Calix[ n ]Arene Skeletons: toward Molecular Recognition and Metal Binding,” Chemical Reviews 97 (1997): 1713–1734, 10.1021/cr960385x. 11851464

[53] Ē. Kupče and T. D. W. Claridge , “NOAH: NMR Supersequences for Small Molecule Analysis and Structure Elucidation,” Angewandte Chemie International Edition 56 (2017): 11779–11783, 10.1002/anie.201705506. 28665502

[54] M. P. Williamson , “Using Chemical Shift Perturbation to Characterise Ligand Binding,” Progress in Nuclear Magnetic Resonance Spectroscopy 73 (2013): 1–16, 10.1016/j.pnmrs.2013.02.001. 23962882

[55] D. Russel , K. Lasker , B. Webb , et al., “Putting the Pieces Together: Integrative Modeling Platform Software for Structure Determination of Macromolecular Assemblies,” PLoS Biology 10 (2012): e1001244, 10.1371/journal.pbio.1001244. 22272186 PMC3260315

[56] M. Isselstein , L. Zhang , V. Glembockyte , et al., “Self‐Healing Dyes—Keeping the Promise?,” Journal of Physical Chemistry Letters 11 (2020): 4462–4480, 10.1021/acs.jpclett.9b03833. 32401520

[57] J. H. M. van der Velde , E. Ploetz , M. Hiermaier , et al., “Mechanism of Intramolecular Photostabilization in Self‐Healing Cyanine Fluorophores,” Chemphyschem 14 (2013): 4084–4093, 10.1002/cphc.201300785. 24302532

[58] J. H. M. van der Velde , J. Oelerich , J. Huang , et al., “A Simple and Versatile Design Concept for Fluorophore Derivatives With Intramolecular Photostabilization,” Nature Communications 7 (2016): 10144, 10.1038/ncomms10144. PMC4729898 26751640

[59] S. Akine and Y. Sakata , “Control of Guest Binding Kinetics in Macrocycles and Molecular Cages,” Chemistry Letters 49 (2020): 428–441, 10.1246/cl.200017.

[60] W. M. Nau and X. Zhang , “An Exceedingly Long‐Lived Fluorescent State as a Distinct Structural and Dynamic Probe for Supramolecular Association: An Exploratory Study of Host–Guest Complexation by Cyclodextrins,” Journal of the American Chemical Society 121 (1999): 8022–8032, 10.1021/ja990626t.

[61] D. Rehm and A. Weller , “Kinetics of Fluorescence Quenching by Electron and H‐Atom Transfer,” Israel Journal of Chemistry 8 (1970): 259–271, 10.1002/ijch.197000029.

[62] T. Evan‐Salem and Y. Cohen , “Octahydroxypyridine[4]Arene Self‐Assembles Spontaneously To Form Hexameric Capsules and Dimeric Aggregates,” European Journal of Chemistry 13 (2007): 7659–7663, 10.1002/chem.200700461. 17623281

[63] E. Persch , O. Dumele , and F. Diederich , “Molecular Recognition in Chemical and Biological Systems,” Angewandte Chemie International Edition 54 (2015): 3290–3327, 10.1002/anie.201408487. 25630692

[64] J. Vogelsang , T. Cordes , and P. Tinnefeld , “Single‐molecule Photophysics of Oxazines on DNA and Its Application in a FRET Switch,” Photochemical & Photobiological Sciences 8 (2009): 486–496, 10.1039/b822318c. 19337662

[65] T. Cordes , J. Vogelsang , and P. Tinnefeld , “On the Mechanism of Trolox as Antiblinking and Antibleaching Reagent,” Journal of the American Chemical Society 131 (2009): 5018–5019, 10.1021/ja809117z. 19301868

