# Supplementary material for: Allosteric Regulation of Photophysics and Binding in Oxazine‐macrocycle Complexes at Single‐molecule Resolution
Source: Angew Chem Int Ed Engl. 2026 May 31;65(29):e1546846. doi: 10.1002/anie.1546846 (PMC13360799; doi:10.1002/anie.1546846)
Supplement: Supplementary file 1 — Supporting File 1: anie72911‐sup‐0001‐SuppMat.docx. [file ANIE-65-e1546846-s001.docx]

**Supporting Information for**

**Allosteric regulation of photophysics and binding in oxazine-macrocycle complexes at single-molecule resolution**

Siyu Lu^1,2,#^, Thomas Peulen^1,#^, Hongbin Wu^1^, Florian Lindemann^3^, Despoina Kapiki^1,2^, Rasmus Linser^3^, Andreas Hennig^4^, and Thorben Cordes^1,2,*^

^1^Biophysical Chemistry, Department of Chemistry and Chemical Biology, Technische Universität Dortmund, Otto-Hahn-Str. 4a, 44227 Dortmund, Germany

^2^Physical and Synthetic Biology, Faculty of Biology, Großhadernerstr. 2-4, Ludwig-Maximilians-Universität München, 82152 Planegg-Martinsried, Germany

^3^Biomolecular NMR Spectroscopy, Department of Chemistry and Chemical Biology, Technische Universität Dortmund, Otto-Hahn-Str. 4a, 44227 Dortmund, Germany

^4^Center for Cellular Nanoanalytics (CellNanOs) and School of Biology / Chemistry, Universität Osnabrück, Barbarastraße 7, 49069 Osnabrück, Germany

#these authors contributed equally to this paper

*corresponding author: [thorben.cordes@tu-dortmund.de](mailto:thorben.cordes@tu-dortmund.de)

**Additional Data and Supplementary Figures**


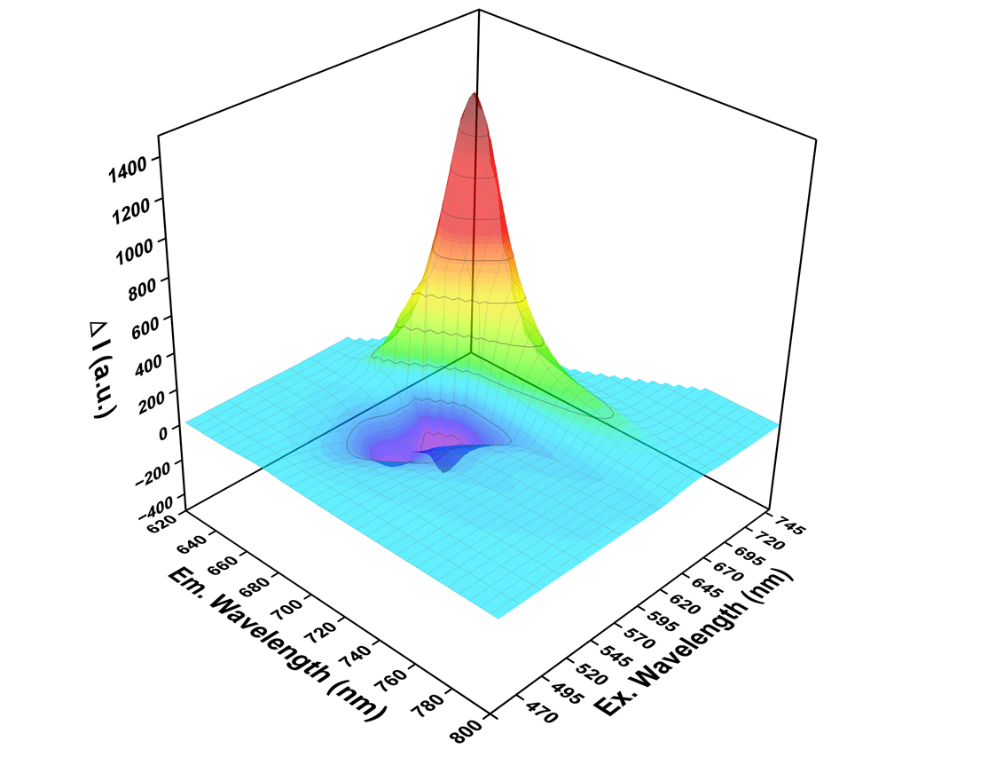


**Figure S1.** Differential excitation-emission matrix showing the spectral shift of ATTO655 upon addition of 25 μM CB8.


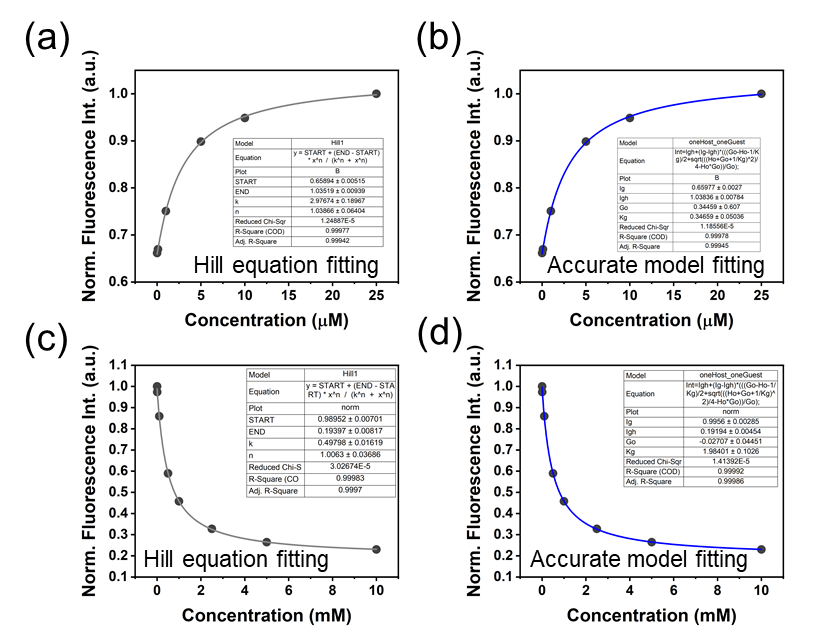


**Figure S2.** Comparison of Hill equation fitting and accurate model fitting for ATTO655 titrated with (a, b) CB8 and (c, d) sCX4. (a) Hill equation binding model with a fitted *K*_d_ of 2.98 μM (*K*_a_ = 0.34 μM^-1^). (b) Accurate binding model fitting yielding a *K*_a_ of 0.35 μM^-1^ (*K*_d_ = 2.88 μM). (c) Hill equation binding model with a fitted *K*_d_ of 0.50 mM (*K*_a_ = 2.01 mM^-1^). (d) Accurate binding model fitting yielding a *K*_a_ of 1.98 mM⁻¹ (*K*_d_ = 0.51 mM). Equations are given in the insert table. Validity of the approximation is given by the fact that the dye concentration is much lower than the *K*_d_.


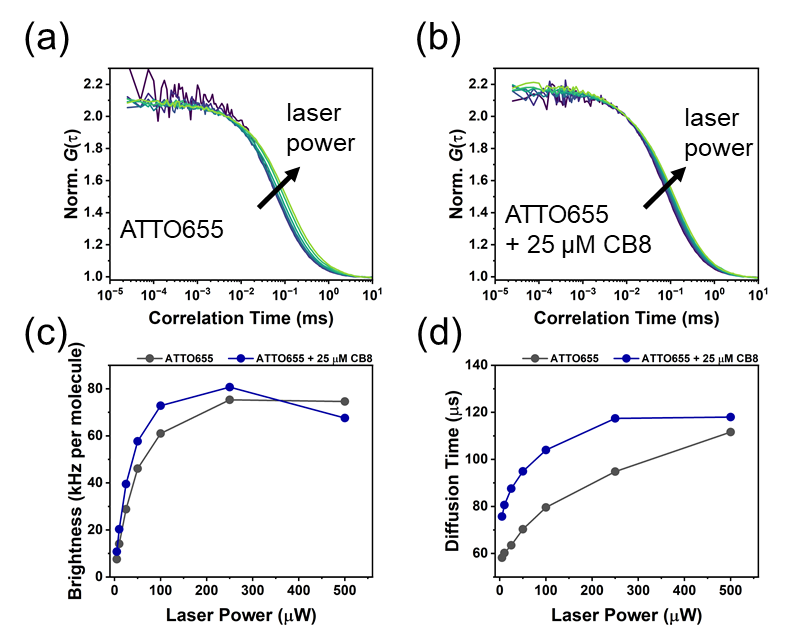


**Figure S3**. FCS analysis of ATTO655 excitation power dependence. Autocorrelation curves of (a) free ATTO 655 and (b) ATTO 655 in the presence of 25 μM CB8 recorded at varying excitation powers. The color gradient from dark to light indicates increasing power. Dependence of (c) molecular brightness and (d) diffusion time on excitation power in the absence and presence of 25 μM CB8. We observed that both the molecular brightness and the diffusion time increase linearly with the laser intensity, but saturate at intensities > 50 µW (17 kW cm^-2^) to ultimately reach a plateau above 200 – 300 µW. This saturation can be attributed to an increasing triplet-state population, seen in an increase of the short bunching term, limiting the available photon count rate to ~70 kHz per molecule for ATTO655 and ~80 kHz for the ATTO655-CB8 complex. This increase in molecular brightness is consistent with the fluorescence increase seen in the spectral analysis in Figure 2. To mitigate any issues related to triplet-state formation and saturation in FCS, the laser power was 25 μW (8.5 kW cm^-2^) for all subsequent experiment of ATTO655, a value below the saturation threshold.


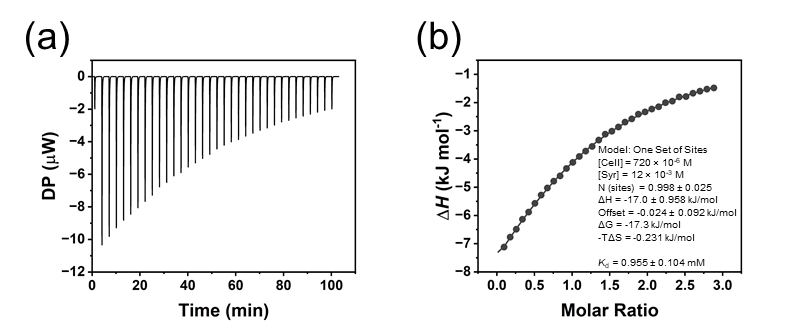


**Figure S4.**ITC analysis of the ATTO655-sCX4 interaction. (a) Raw ITC thermogram. (b) Integrated heat data plotted versus molar ratio (*K*_a_ = (1.00 ± 0.07) × 10^3^ M^-1^, *K*_d_ = 1.00 mM, n = 3; the solid line indicates fit). Concentration: 0.72 mM ATTO655 (cell) and 12 mM sCX4 (syringe). To compensate for the low affinity of the interaction, sCX4 was used as the titrant at a 16.7-fold higher concentration over ATTO655. The representative curve is shallow, demonstrating a typical titration with a low Wiseman constant. The fitted binding stoichiometry (*N*) was 1.01 ± 0.01 and suggests a 1:1 binding interaction.


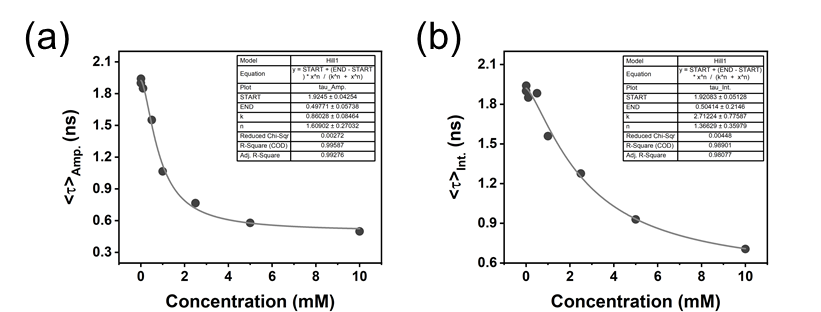


**Figure S5.** Mean fluorescence lifetimes of ATTO655 as a function of sCX4 concentration. (a) Amplitude-weighted mean lifetime (⟨τ⟩_Amp._) and (b) intensity-weighted mean lifetime (⟨τ⟩_Int._).


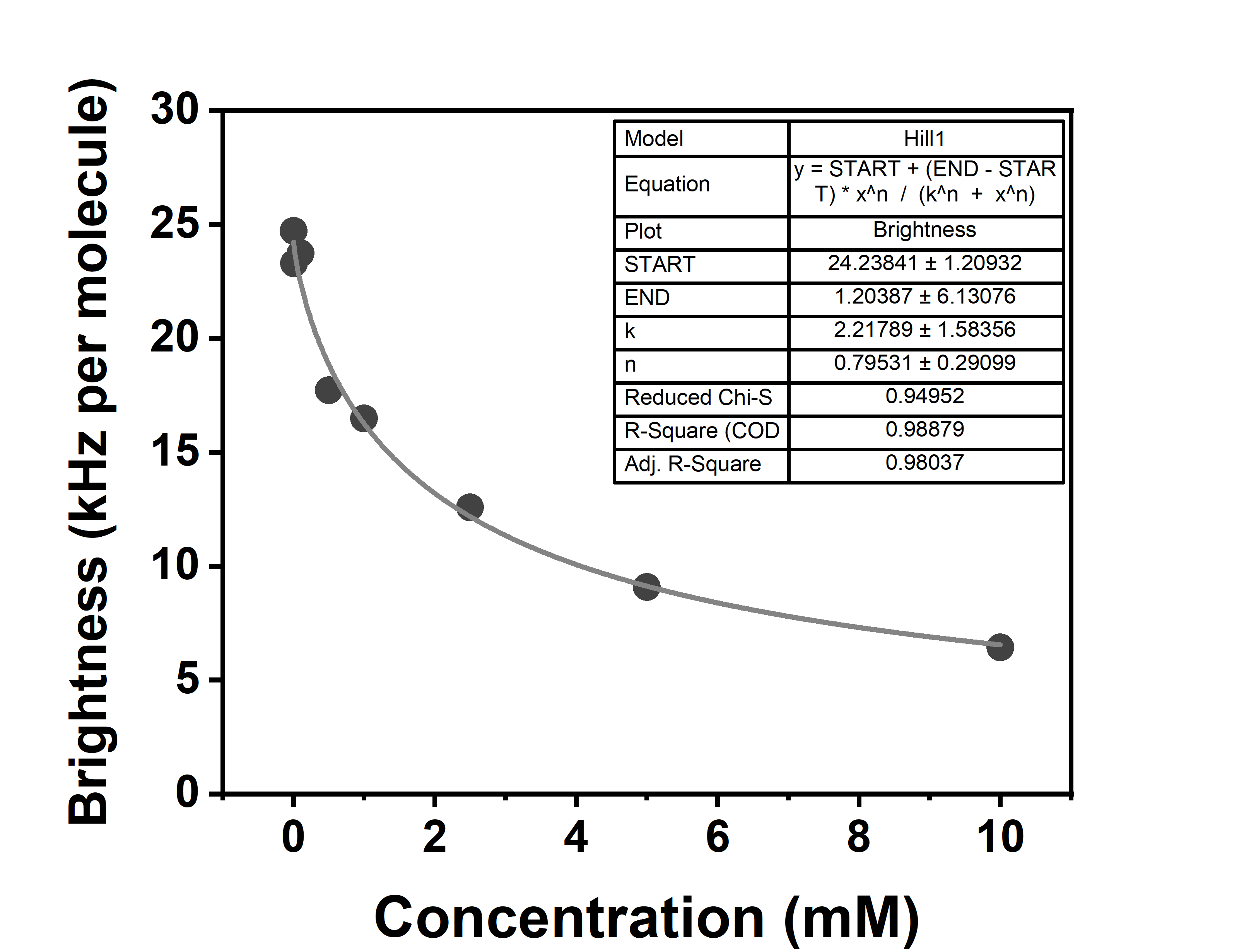


**Figure S6.** Molecular brightness of ATTO655 based on FCS experiments as a function of sCX4 concentration.


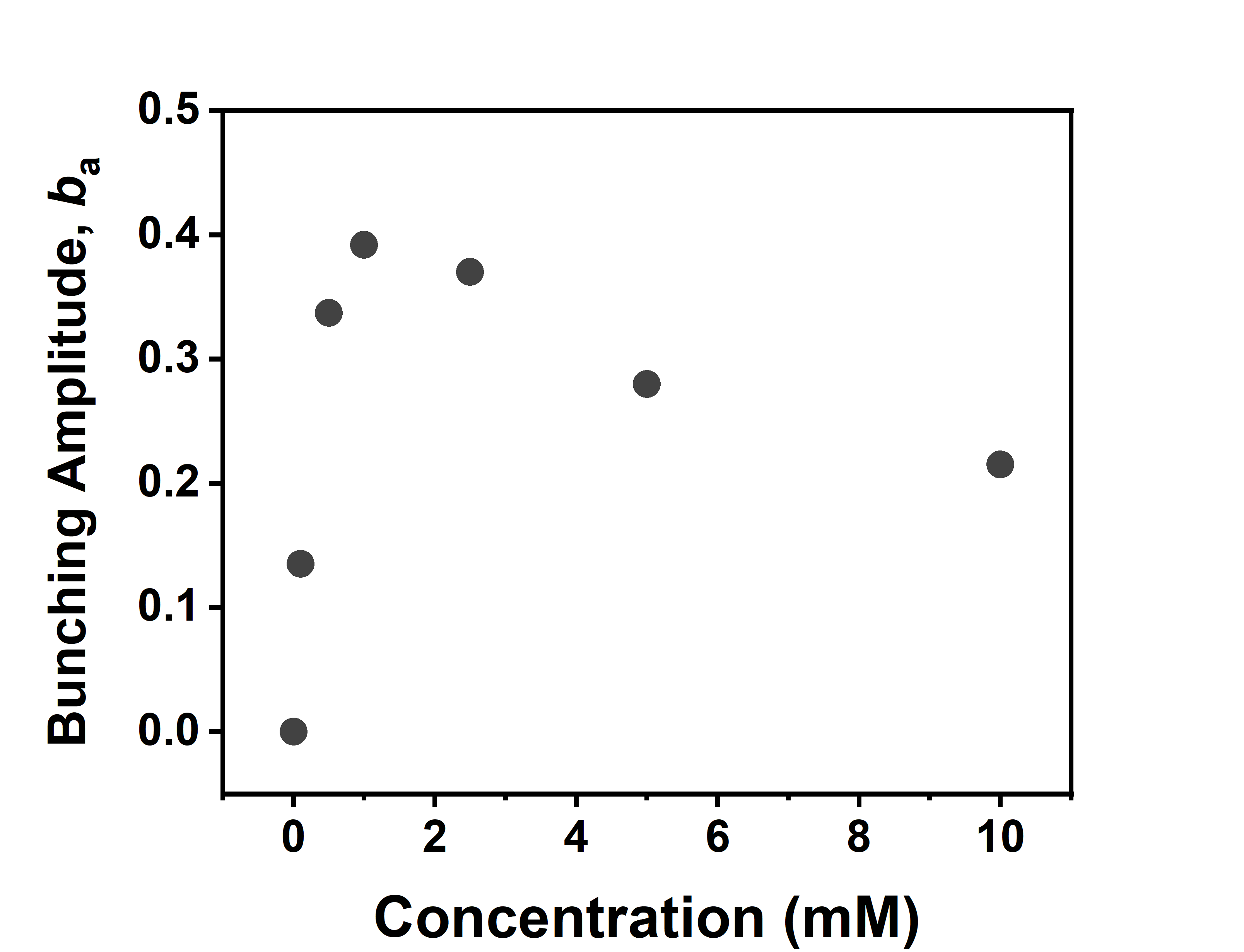


**Figure S7.** Bunching amplitude of ATTO655 based on FCS experiments as a function of sCX4 concentration.


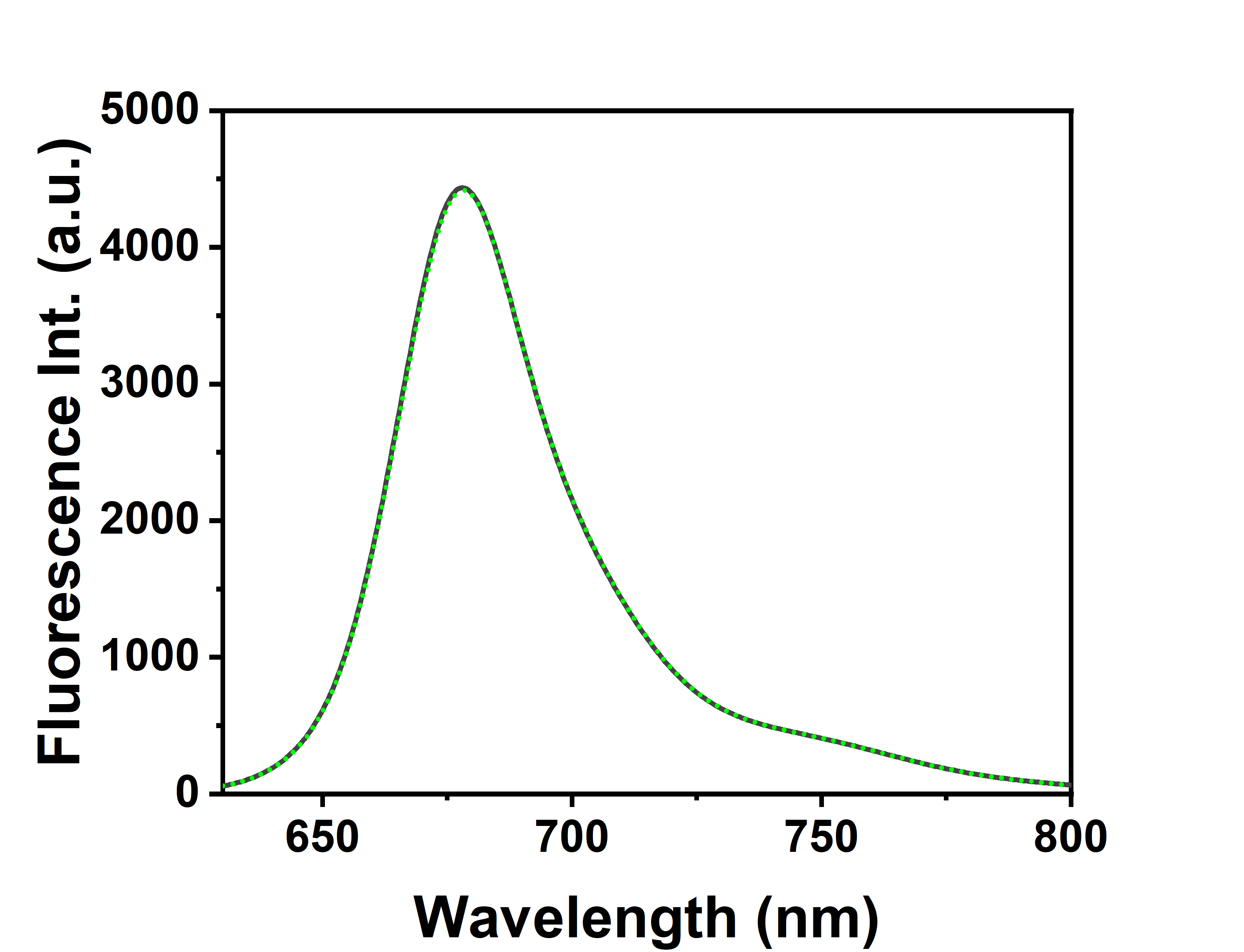


**Figure S8.** Effect of choline on ATTO655 fluorescence. Emission spectra of ATTO655 in the absence (black solid line) and presence (green dashed line) of 10.5 mM choline. The identical spectra confirm that choline does not impact the fluorescence properties of ATTO655.


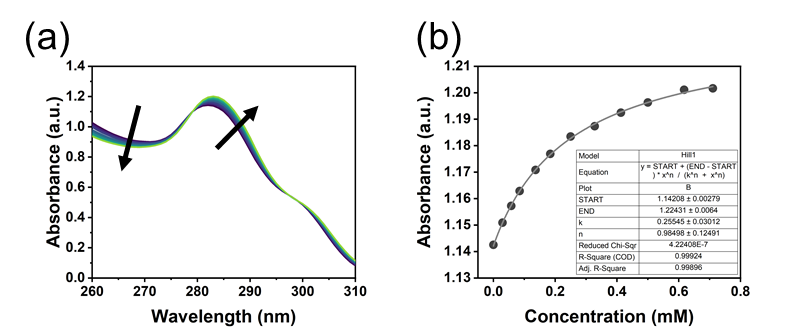


**Figure S9. (a)** Absorbance titration spectra of sCX4. The color gradient indicates increasing concentration of choline from dark to light. (b) Maximum absorbance intensity (260 – 310 nm) of sCX4 plotted as a function of choline concentration. The grey line represents a data fit yielding a *K*_a_ of 3.9 × 10^3^ M^-1^ (*K*_d_ = 0.26 mM).


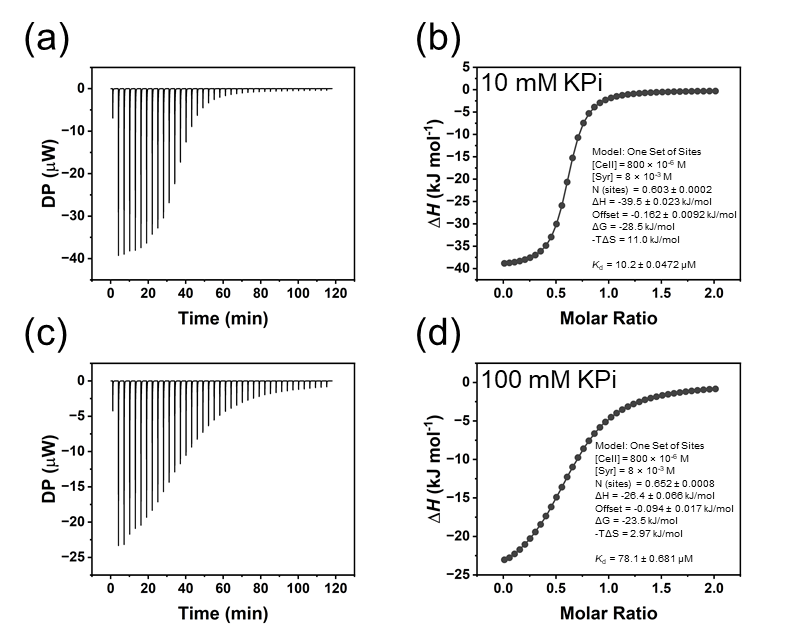


**Figure S10.** ITC analysis of the choline-sCX4 interaction in (a, b) 10 mM and (c, d) 100 mM KP_i_ buffer (pH7.6). Panels (a, c) show the raw ITC thermogram. Panels (b, d) display the integrated heat data plotted against molar ratio; the solid lines indicate the best fit to the data. Association constants were determined to be (b) 1.0 × 10^5^ M^-1^ (*K*_d_ = 10 μM) and (d) 1.3 × 10^4^ M^-1^ (*K*_d_ = 78 μM). The used concentrations were 0.8 mM of sCX4 (cell) and 8 mM choline (syringe).


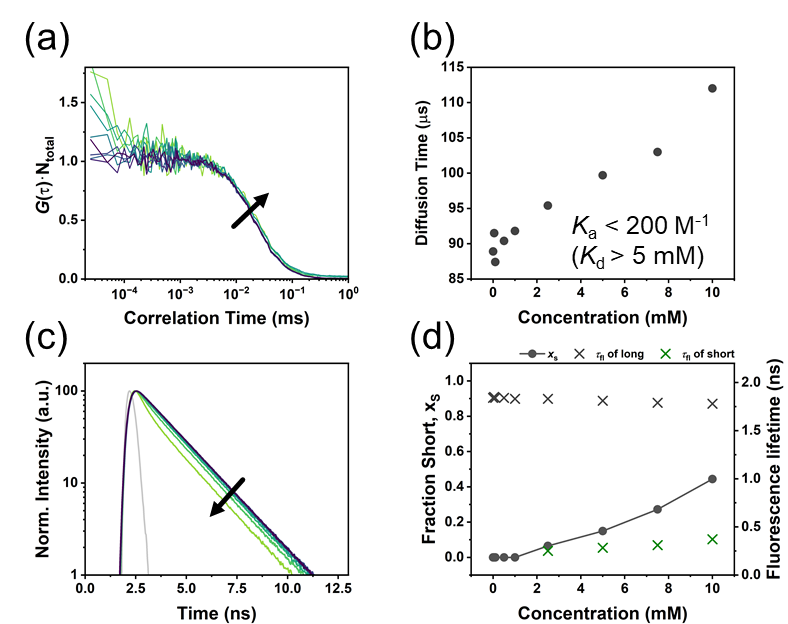


**Figure S11.** Diffusion time and fluorescence lifetime analysis of ATTO655 in the presence of 12 mM choline and varying concentrations of sCX4 (0 – 10 mM). (a) FCS curves. (b) Diffusion times derived from FCS. (c) Normalized time-correlated single-photon counting (TCSPC) fluorescence decay curves. (d) Fluorescence lifetime analysis showing the fraction of the short lifetime component and the lifetime values of the short and long components. The color gradient from dark to light indicates increasing sCX4 concentrations. Experimental conditions: 640 nm excitation, 25 μW (5.3 kW cm^-2^), water objective.

**
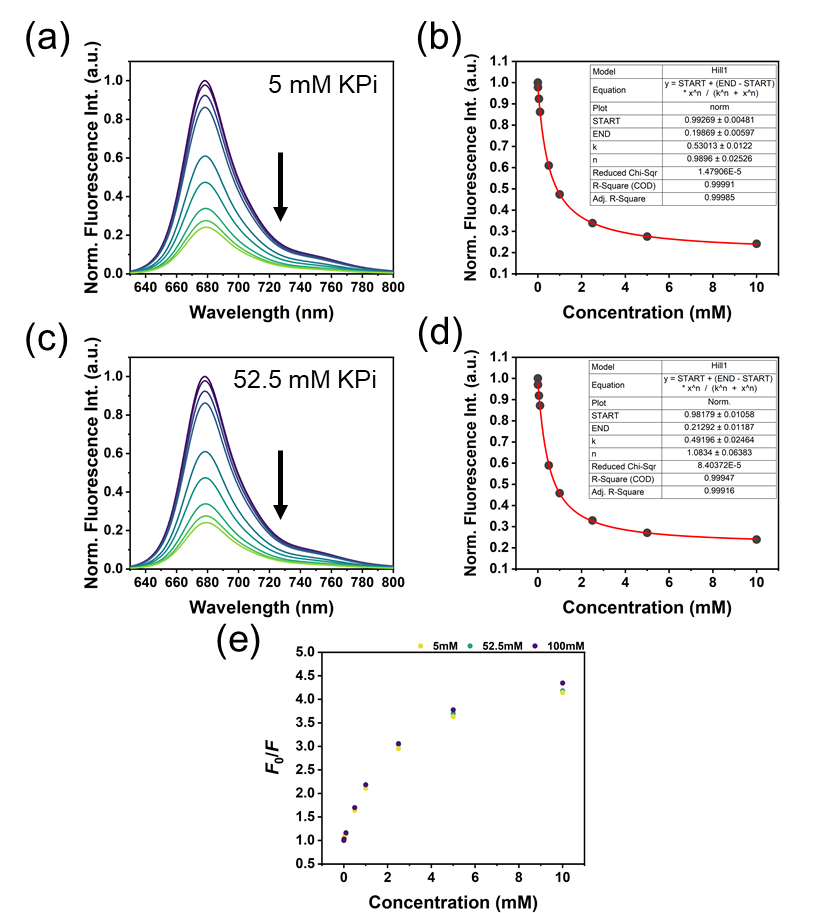
**

**Figure S12.** Fluorescence titration of ATTO655 with sCX4 at varying buffer concentrations. (a) Fluorescence spectra in 5 mM KP_i_ (pH 7.4) and (b) the corresponding data fit (*K*_d_ = 0.53 mM). (c) Fluorescence spectra in 52.5 mM KP_i_ (pH 7.4) and (d) the corresponding data fit (*K*_a_ = 2.04 × 10^3^ M^-1^; *K*_d_ = 0.49 mM). (e) Stern-Volmer overlay for 5, 52.5, 100 mM KP_i_ concentrations. The 100 mM data is identical to that shown in Figure 4.

**
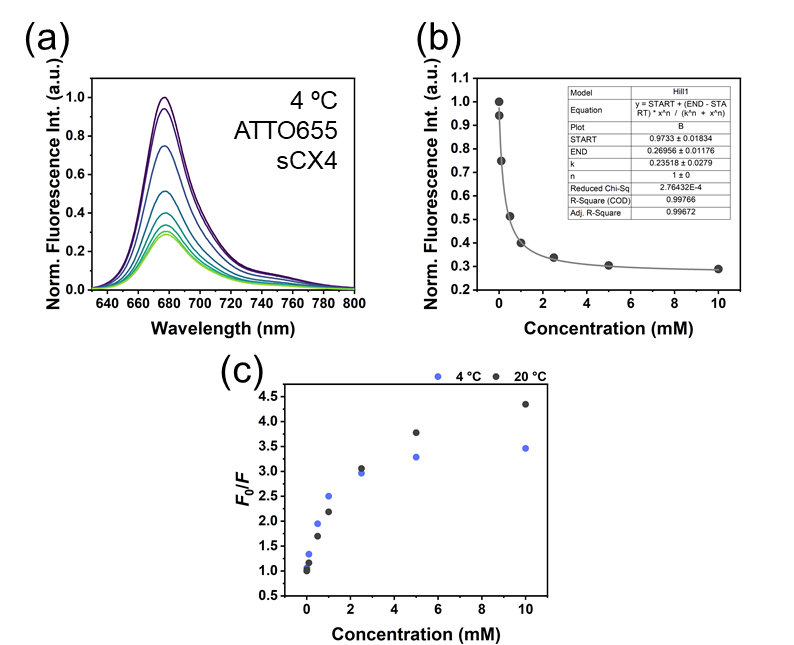
**

**Figure S13.** Fluorescence titration of ATTO655 with sCX4 (0 – 10 mM, color gradient from dark to light indicates increasing concentration) at 4 ºC. (a) Normalized fluorescence spectra and (b) corresponding fluorescence intensity of ATTO655 plotted against sCX4 concentration with the results of fit (grey line; *K*_a_ = 4.2 × 10^3^ M^-1^; *K*_d_ = 0.24 mM). (c) Stern-Volmer overlay plot comparing data at 4 ºC and 20 ºC. The 20 ºC data is reproduced from Figure S5b.


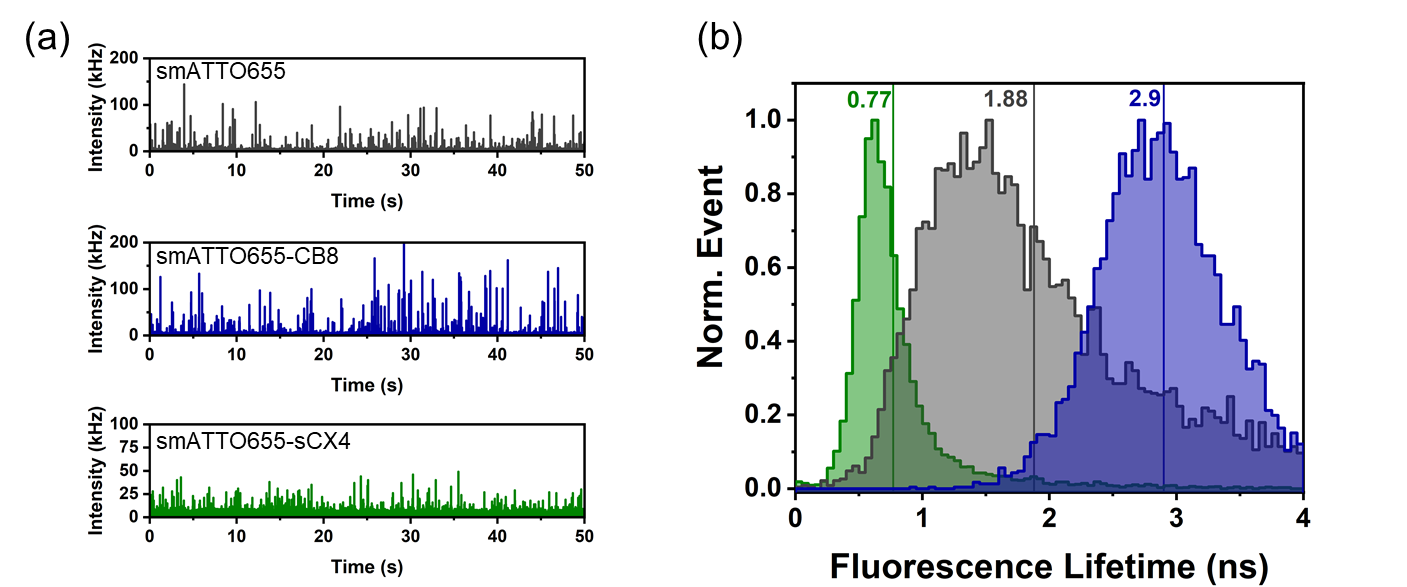


**Figure S14.** Results of single-molecule experiments on freely diffusing ATTO655. (a) Fluorescence intensity traces and (b) normalized histograms showing the distribution of fluorescence lifetimes. Grey: ATTO655, blue: ATTO655 in addition of 25 µM of CB8 and green: ATTO655 in addition of 2 mM of sCX4. Laser: 150 µW, 640 nm excitation. The mean fluorescence lifetime of ATTO655, ATTO655-CB8 and ATTO655-sCX4 are 0.77, 1.88 and 2.90 ns, respectively.

**
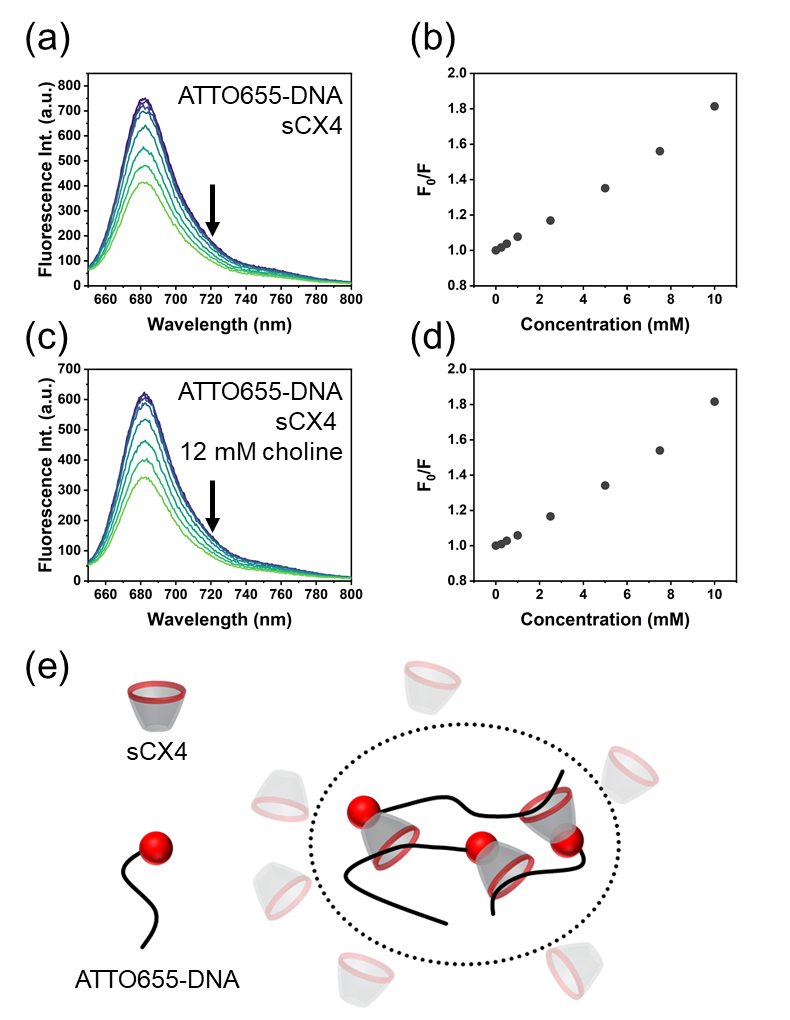
**

**Figure S15.** Fluorescence emission spectra and Stern-Volmer analysis of ATTO655-dsDNA titrated with sCX4 (0 – 10 mM). Data are shown (a, b) in the absence and (c, d) in the presence of 12 mM choline. The color gradient from dark to light indicates increasing sCX4 concentration. The analysis indicates dominant static quenching with low affinity with a slight upward trend of the Stern-Volmer curve that suggesting relevance of dynamic quenching at concentrations of sCX4 > 1 mM.

**
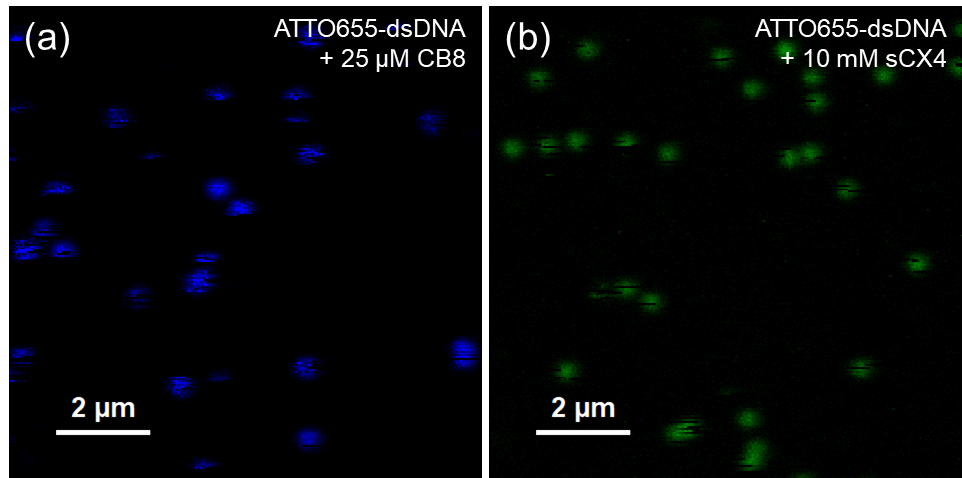
**

**Figure S16**. Representative FLIM images of immobilized ATTO655-dsDNA in presence of (a) 25 μM CB8 and (b) 10 mM sCX4 under 5 μW 640 nm excitation.


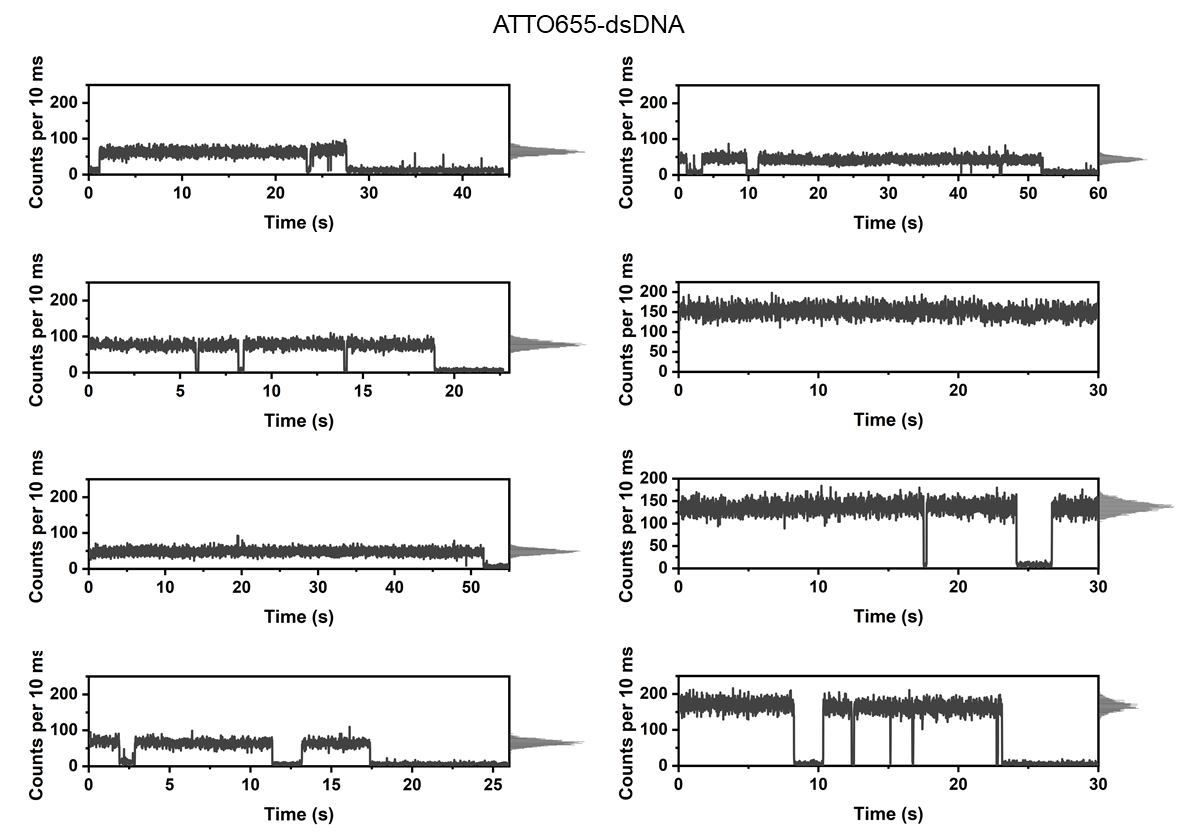


**Figure S17.** Extended dataset showing representative fluorescence time traces of ATTO655-dsDNA obtained under the same conditions as in Figure 6a.


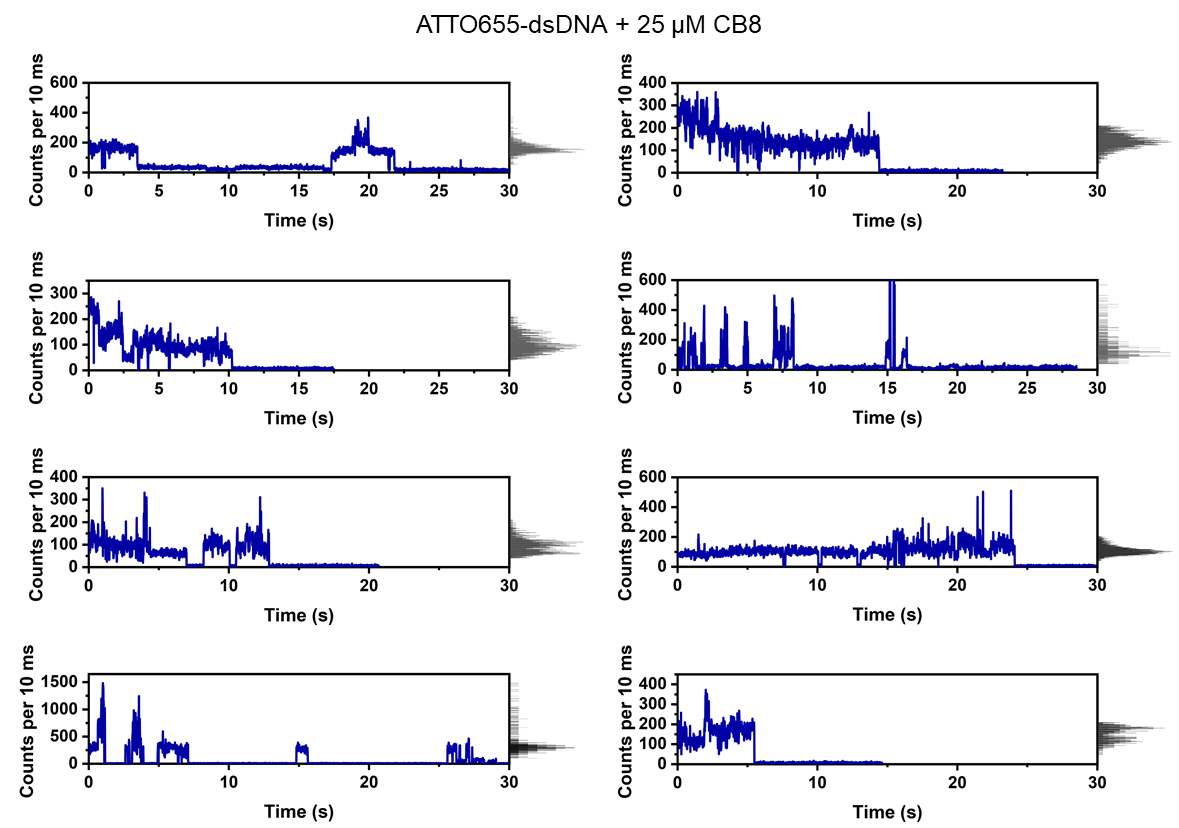


**Figure S18.** Extended dataset showing representative fluorescence time traces of ATTO655-dsDNA obtained under the same conditions as in main text Figure 7a (in addition of 25 μM CB8).


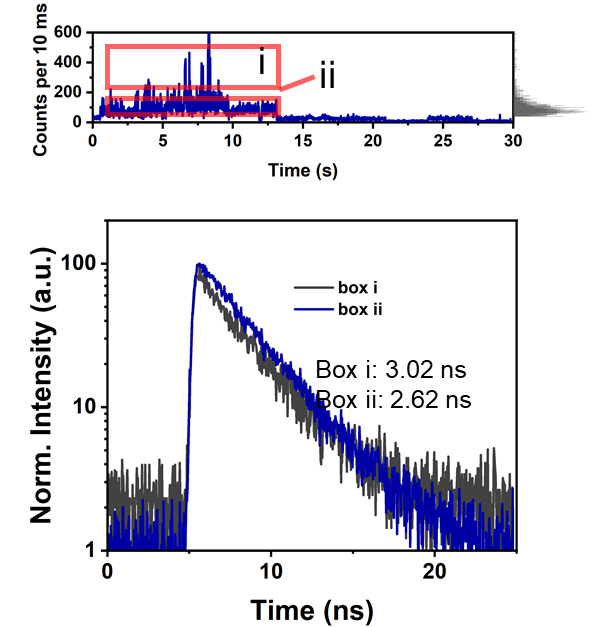


**Figure S19.** Fluorescence lifetime decay profiles extracted from selected windows of the fluorescence time trace is indicated in the upper panel.


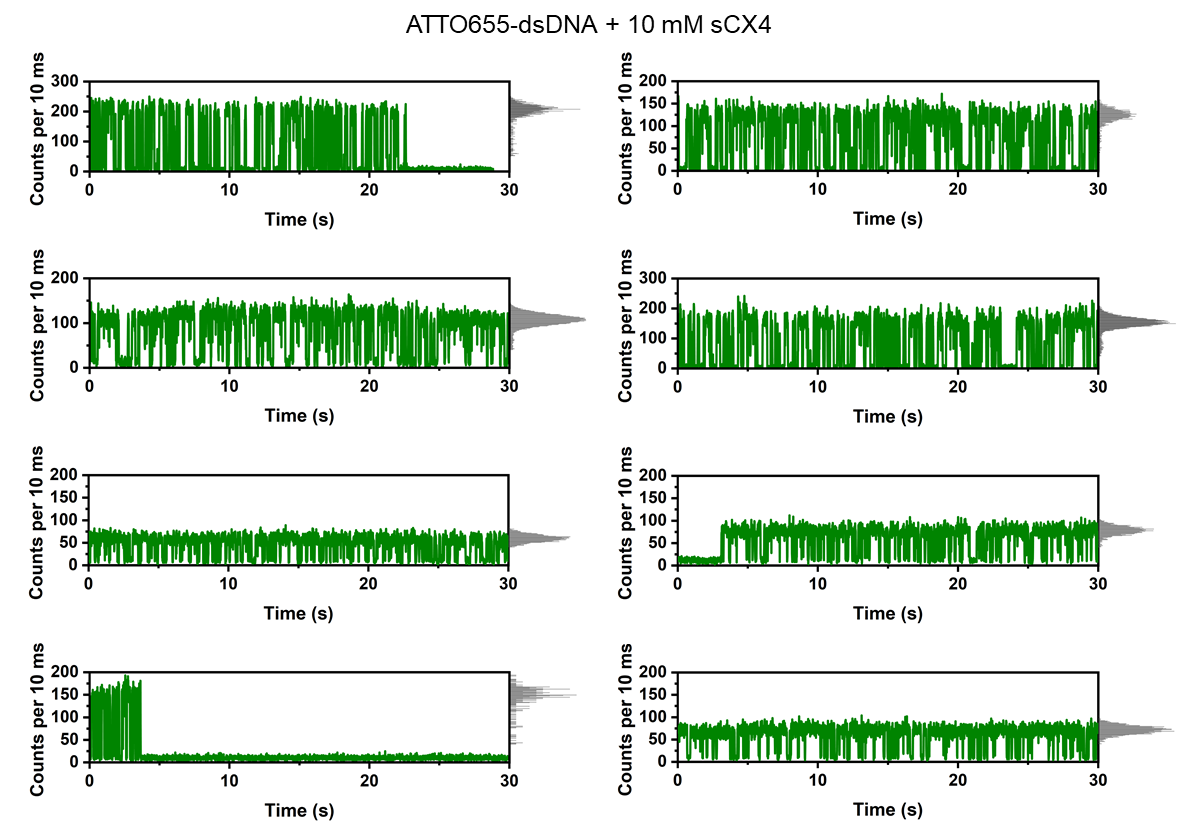


**Figure S20.** Extended dataset showing representative fluorescence time traces of ATTO655-dsDNA obtained under the same conditions as in Figure 7b (in addition of 10 mM sCX4).


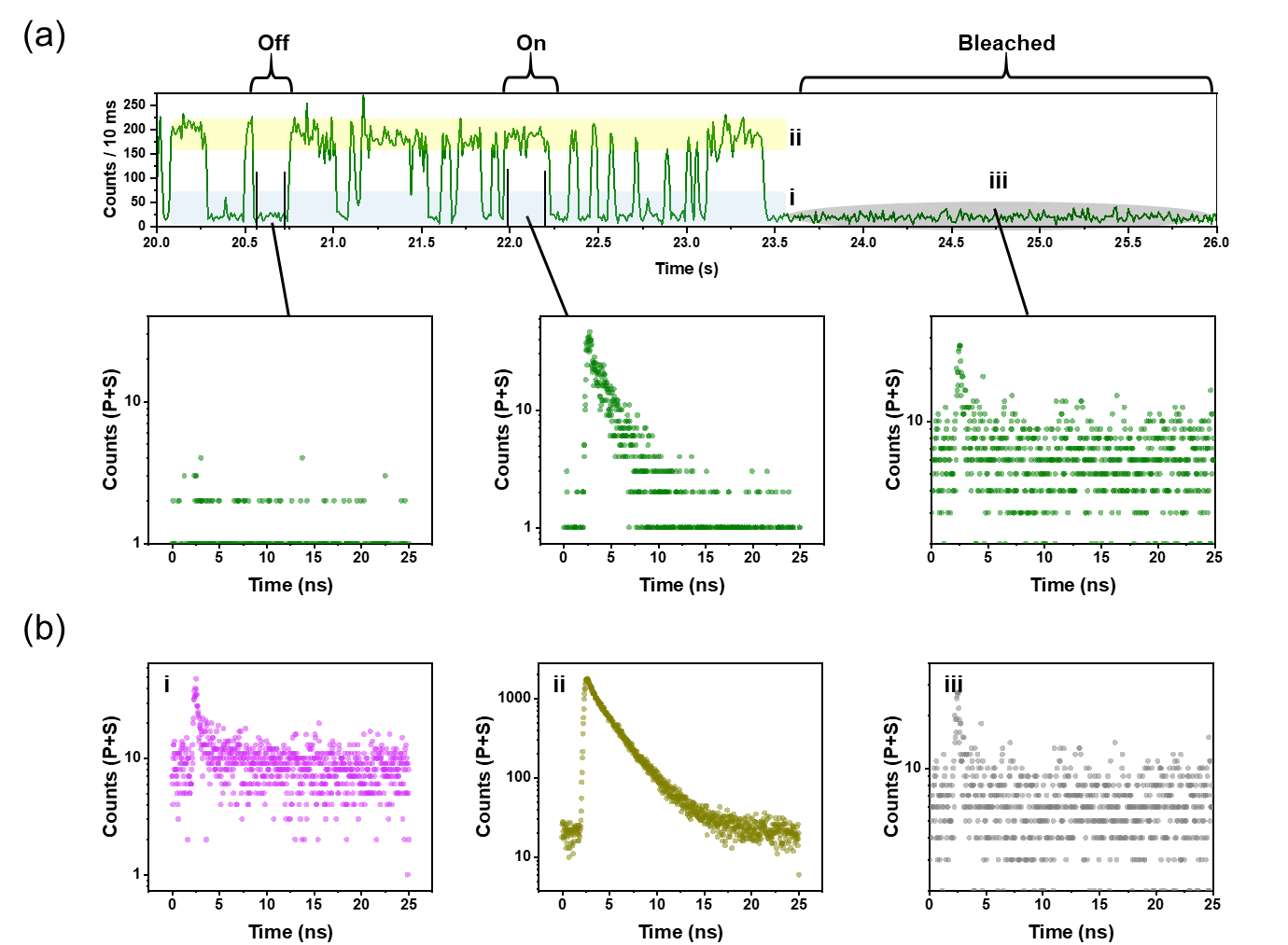


**Figure S21.** (a) Zoomed-in fluorescence trace segments highlighting the corresponding off-state, on-state, and post-bleaching regions (b) Accumulated fluorescence lifetime decays constructed from photons in region i (off-state), ii (on-state) and iii (bleached state).


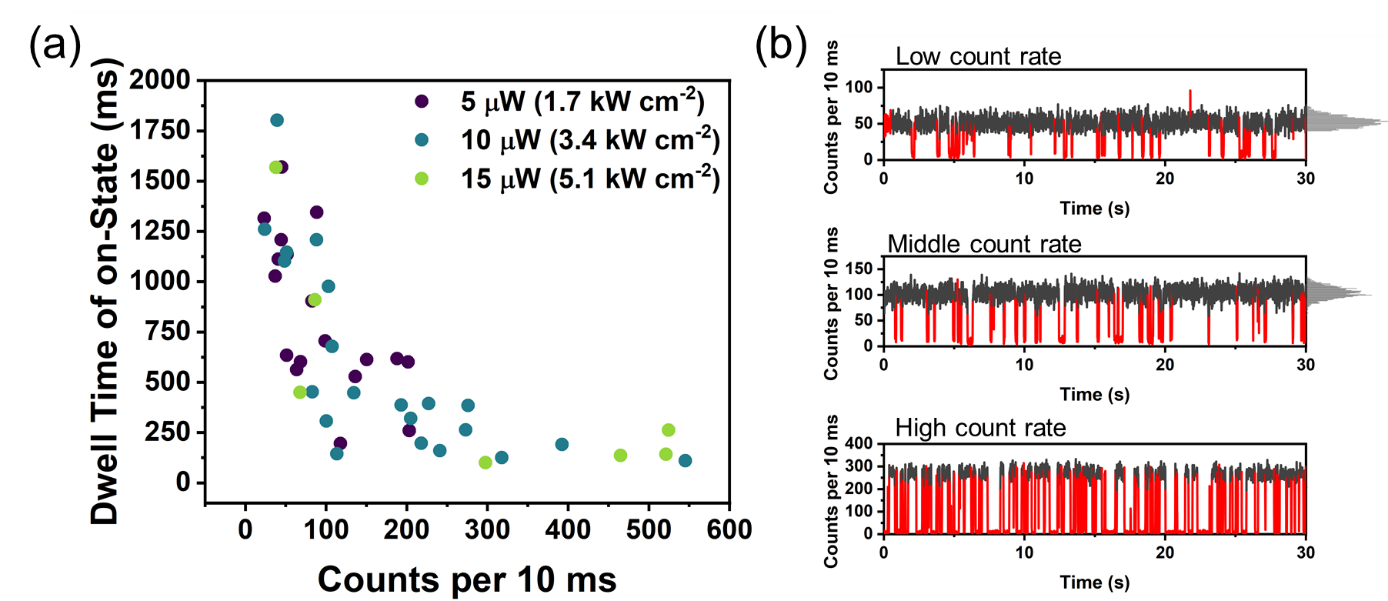


**Figure S22.** (a) Relationship between the on-state dwell time and photon count rate for immobilized ATTO655-dsDNA in the presence of 2.5 mM sCX4. (b) Representative fluorescence time traces with off-states highlighted.


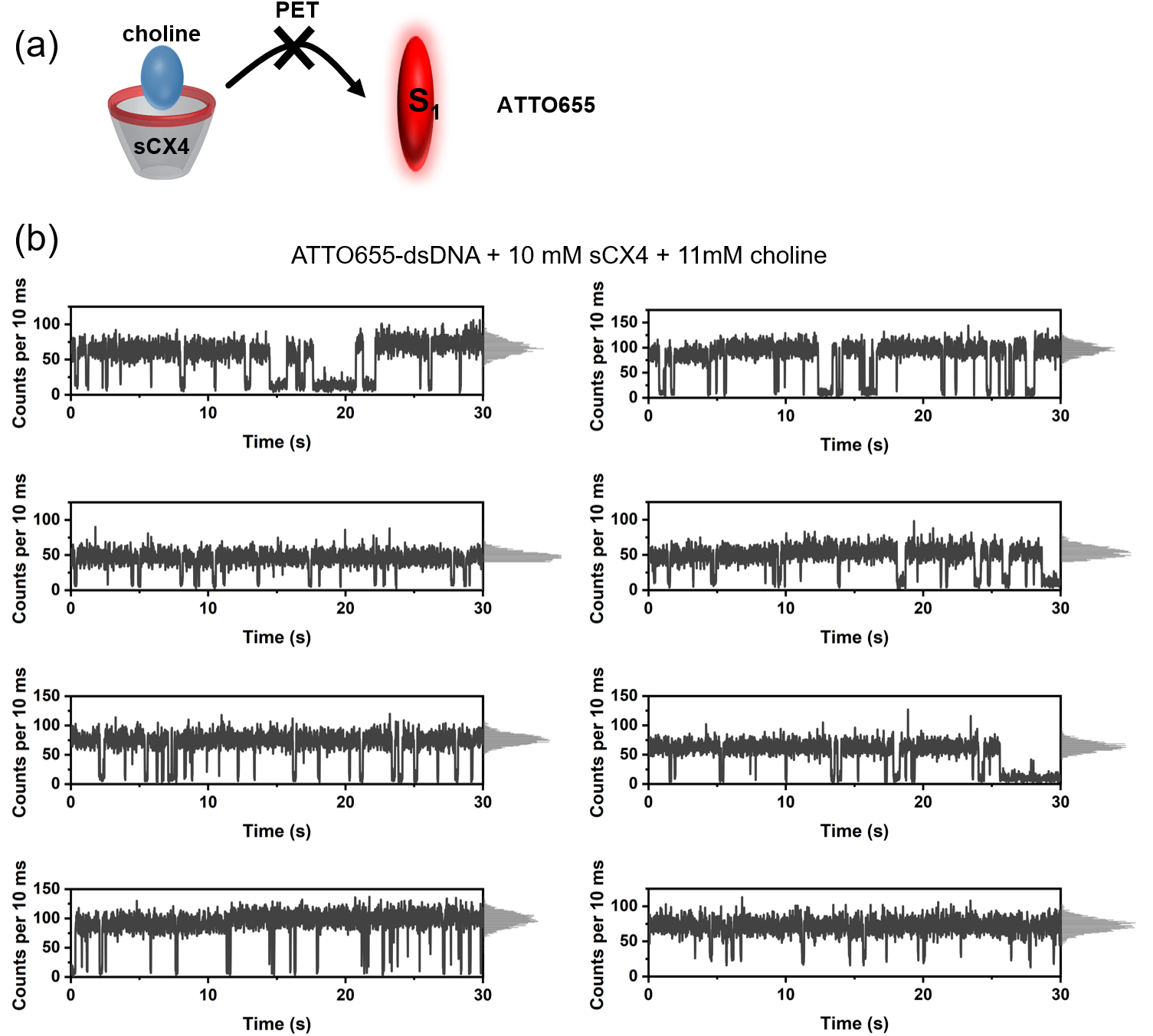


**Figure S23.** (a) Schematic illustration showing reduced photo-induced electron transfer (PET) of the sCX4–choline complex toward ATTO655. (b) Representative fluorescence time traces of immobilized ATTO655-dsDNA in the presence of 10 mM sCX4 and 11 mM choline. Excitation: 640 nm, 5 µW (1.7 kW cm^-2^).

**
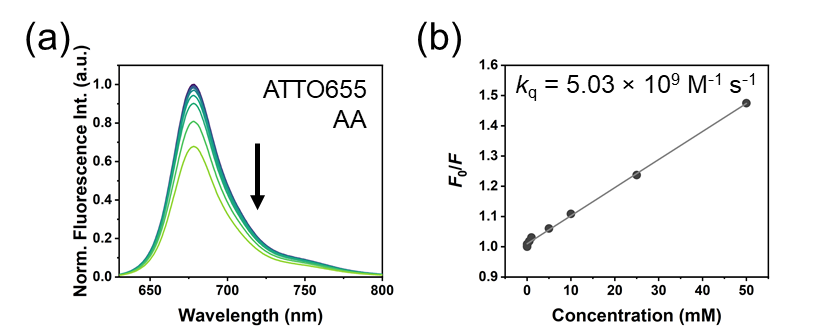
**

**Figure S24.** Fluorescence titration of ATTO655 with ascorbic acid (AA; 0–50 mM in 100 mM KP_i_ buffer). (a) Normalized fluorescence spectra; the color gradient from dark to light indicates increasing AA concentration. (b) Stern-Volmer plot yielding a dynamic quenching constant of *k*_q_ = 5.03 × 10^9^ M^-1^ s^-1^ near the diffusion-limited case.


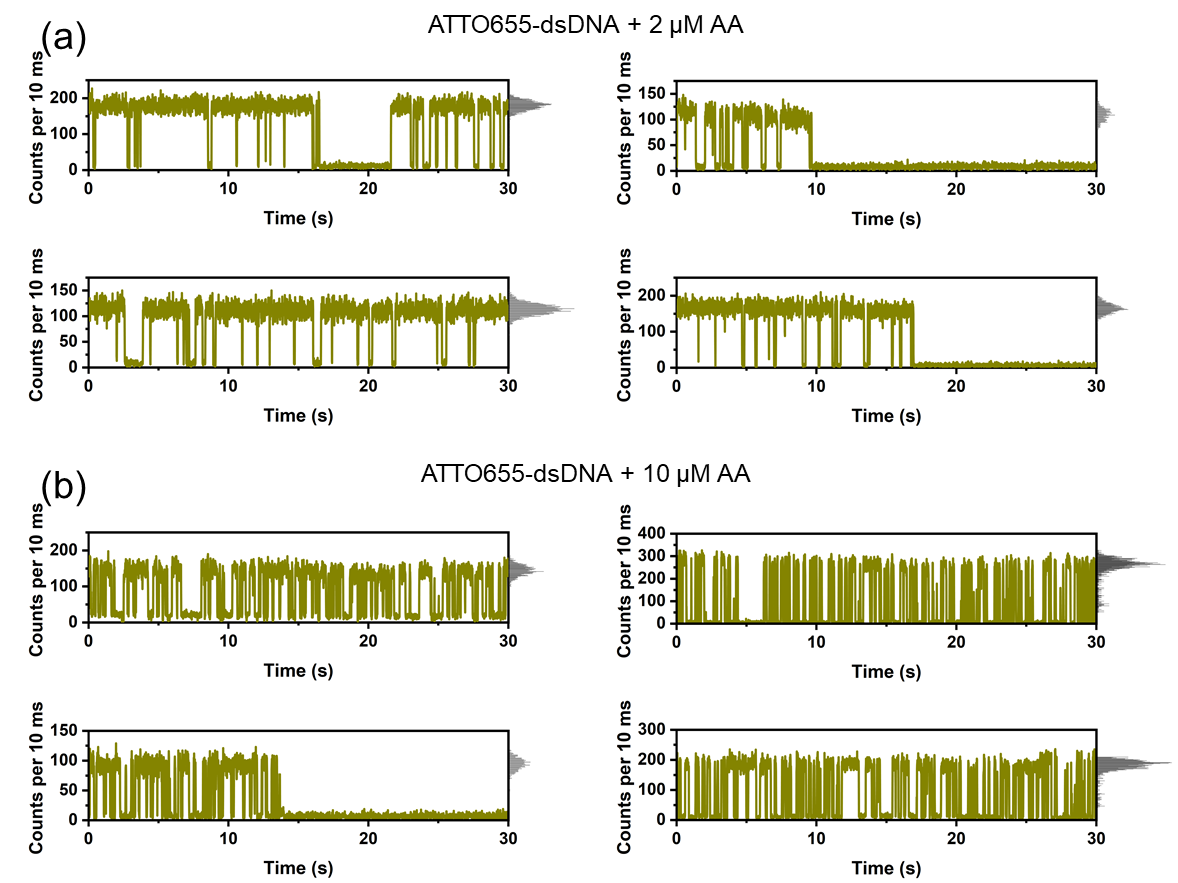


**Figure S25.** (a) Representative fluorescence time traces of immobilized ATTO655-dsDNA in the presence of 2 µM and (b) 10 µM AA. Excitation: 640 nm, 5 µW (1.7 kW cm^-2^).


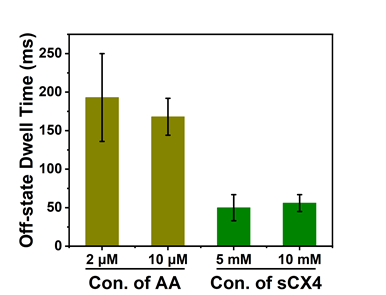


**Figure S26.** Off-state dwell times of ATTO655-dsDNA in the presence of AA or sCX4. The sCX4 data are reproduced from Figure 6e for comparison. No significant difference was observed between 2 µM and 10 µM AA (*p* > 0.05) or between 5 mM and 10 mM sCX4 (*p* > 0.05).


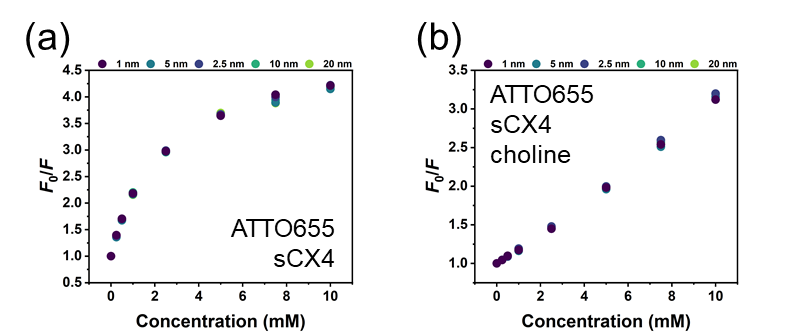


**Figure S27.** Stern-Volmer analysis for ATTO655 with sCX4 (a) without and (b) with 12 mM choline, measured at different excitation slit widths.

**Supplementary Note 1: NMR spectroscopy and structural modelling of the ATTO655-sCX4 complex**

To assess the binding mode and geometry of the ATTO655-sCX4 complex we conducted various NMR experiments combined with structural modelling of the complex using NOESY and CSP information as constraints.

**NMR spectroscopy**

In order to determine atom-specific chemical shifts, and to characterize the interaction between ATTO655 and sCX4 (Figure S28), a series of NMR experiments were recorded. 1D ^1^H experiments were acquired with and without presaturation to remove the signal of residual water (~4.7 ppm) in the D_2_O (Figure S29). However, the use of water presaturation affects adjacent signals, which leads to incorrect intensities and integrals. We note that signal integration is only reliable when the residual water signal is not removed.


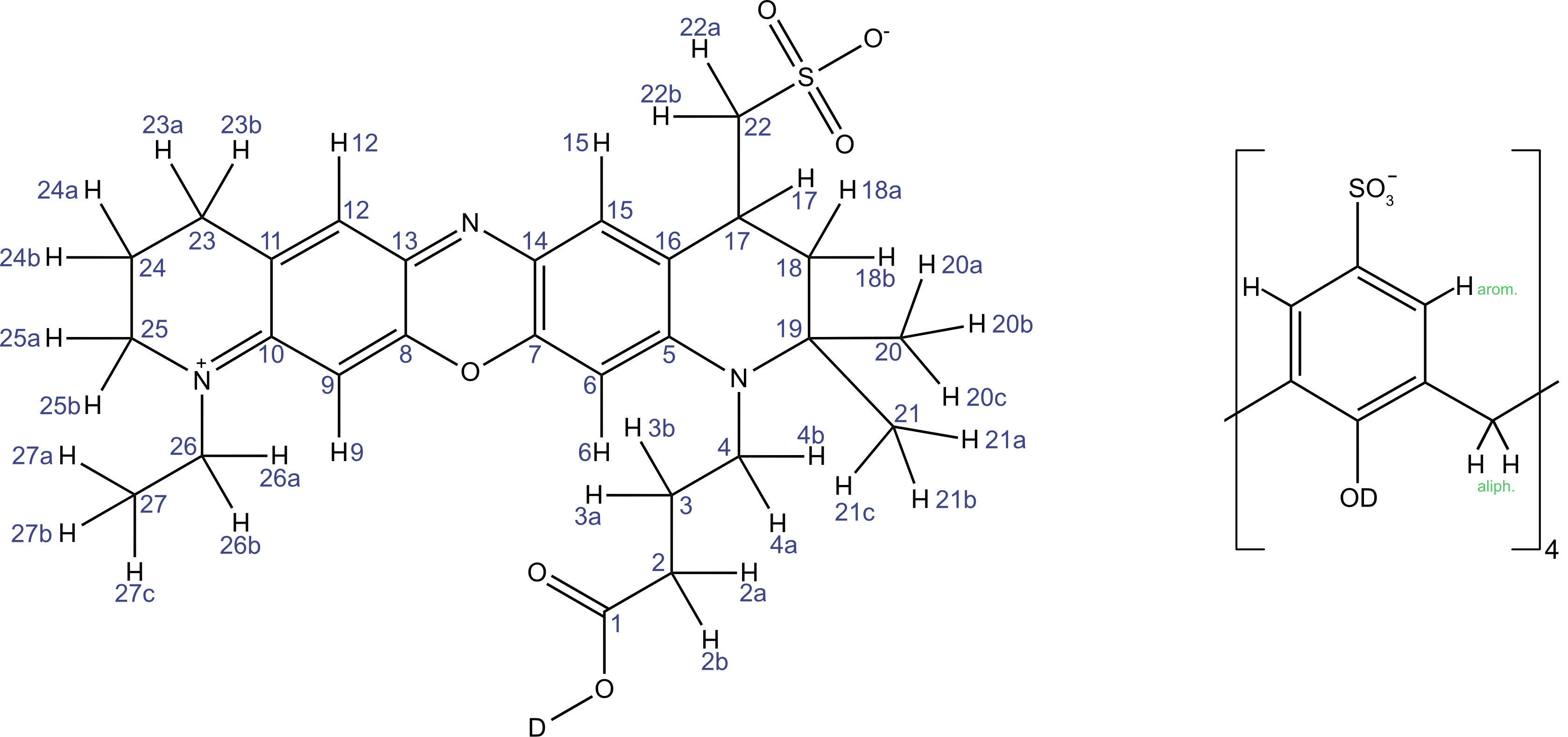


**Figure S28.** Lewis structures of ATTO655 (left) and *p*-sulfonatocalix[4]arene (sCX4, right) with the atom designation used in this publication.

For the assignment, spectra from the manufacturer-provided pulse sequences *noah4_BSCN*[^1^] and *noesyfpgpphwg*[^2^] were used. The *noah4_BSCN* sequence yields four spectra (HMBC, HSQC, COSY, NOESY) and *noesyfpgpphwg* was run to achieve a NOESY spectrum with better resolution. Crucially, the HSQC was acquired with multiplicity editing that allowed for distinction of CH_2_ (positive signal amplitude) from CH/CH_3_ (negative signal amplitude). This enabled direct identification of the only non-aromatic CH group in ATTO655, residing at position 17 (compare Figure S30 and Figure S31 at δ(^1^H) = 3.2 ppm). The second starting point was the methyl group in position 27 which, in contrast to the two others, shows a distinct triplet coupling pattern due to the neighboring CH_2_ (position 26). From those two “anchors”, information from COSY (yielding neighboring ^1^H shifts), HMBC (giving shifts for multi-bond adjacent carbons), and NOESY (^1^H in spatial proximity) spectra could be combined to discern all proton and most carbon chemical shifts. Interestingly, only the left part of the molecule (carrying the carboxy- and sulfonic-acid substituents) gave peaks in the HMBC. The side carrying the ethyl substituent only yielded one HMBC correlation between the aromatic CH sitting at position 12 and the carbon at position 23. A list of assignments can be found in Table S1. Carbon chemical shifts for sCX4 were not assigned. The sCX4-containing samples exhibited a minor impurity encompassing at least one proton and two carbon chemical shifts (possibly acetone). The ATTO655 ^1^H signals at positions 2, 24, 25, 26, and 27 experience significant line broadening when the dye interacts with sCX4. This could arise from multiple interchanging conformational states/binding poses in the complex or increased *T*_2_ relaxation due to association/dissociation dynamics in the presence of the aromatic π-system of sCX4.

The assignment data, Pymol files of ATTO655, and the CcpNmr3 AnalysisAssign [^3^] project together with the raw and processed NMR data are deposited to Zenodo (<https://doi.org/10.5281/zenodo.18939257>).


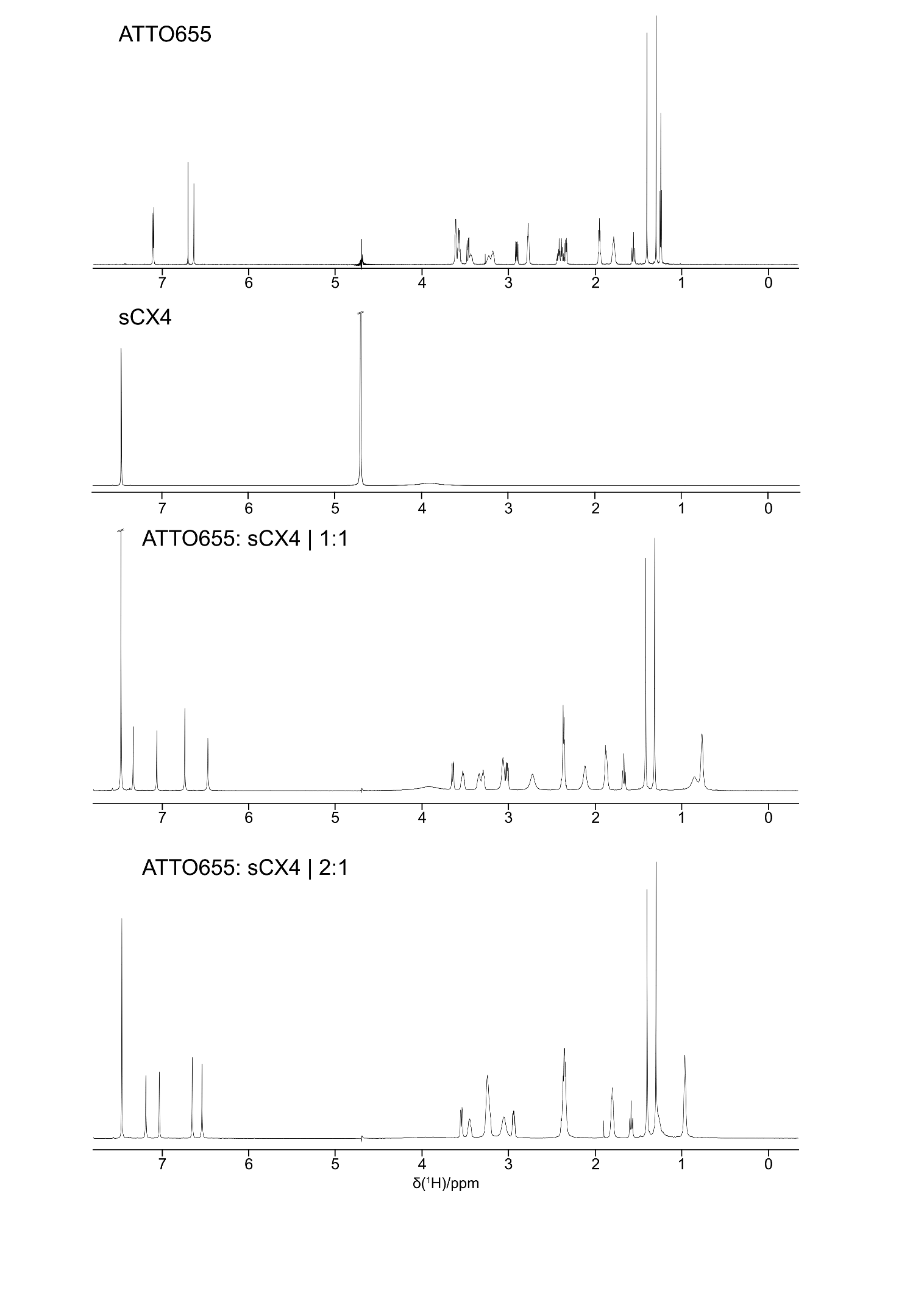
**Figure S29.** 1D ^1^H spectra of ATTO655, sCX4, and their mixtures. The sCX4 spectrum was recorded without water suppression and exhibits three peaks (H_arom._ at 7.4 ppm, H_ali._ at 3.9 ppm and residual water at 4.7 ppm). All other spectra were acquired with water suppression by presaturation during the recycle delay.


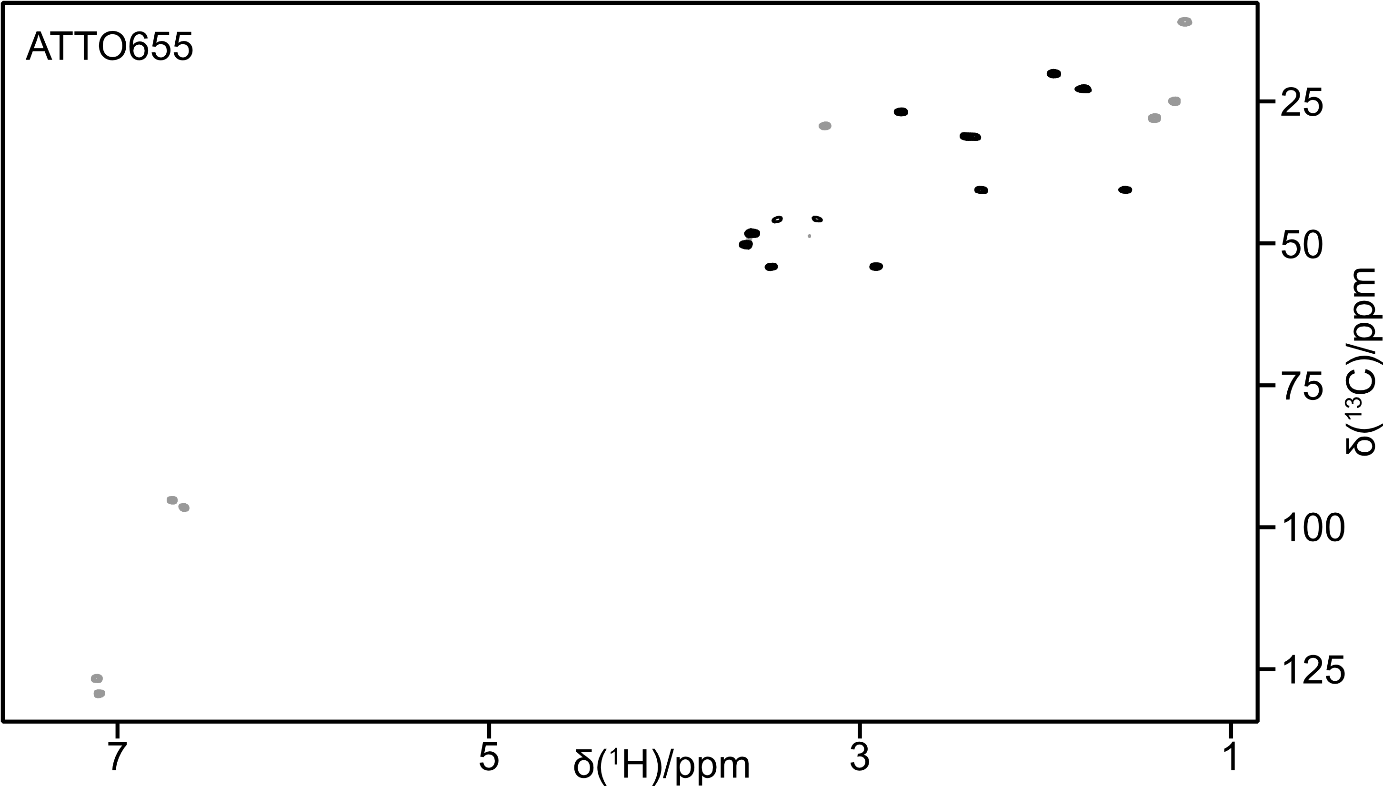


**Figure S30.** 2D ^1^H-^13^C HSQC with multiplicity editing recorded on ATTO655. Positive amplitude signals are shown in black, negative ones in gray.


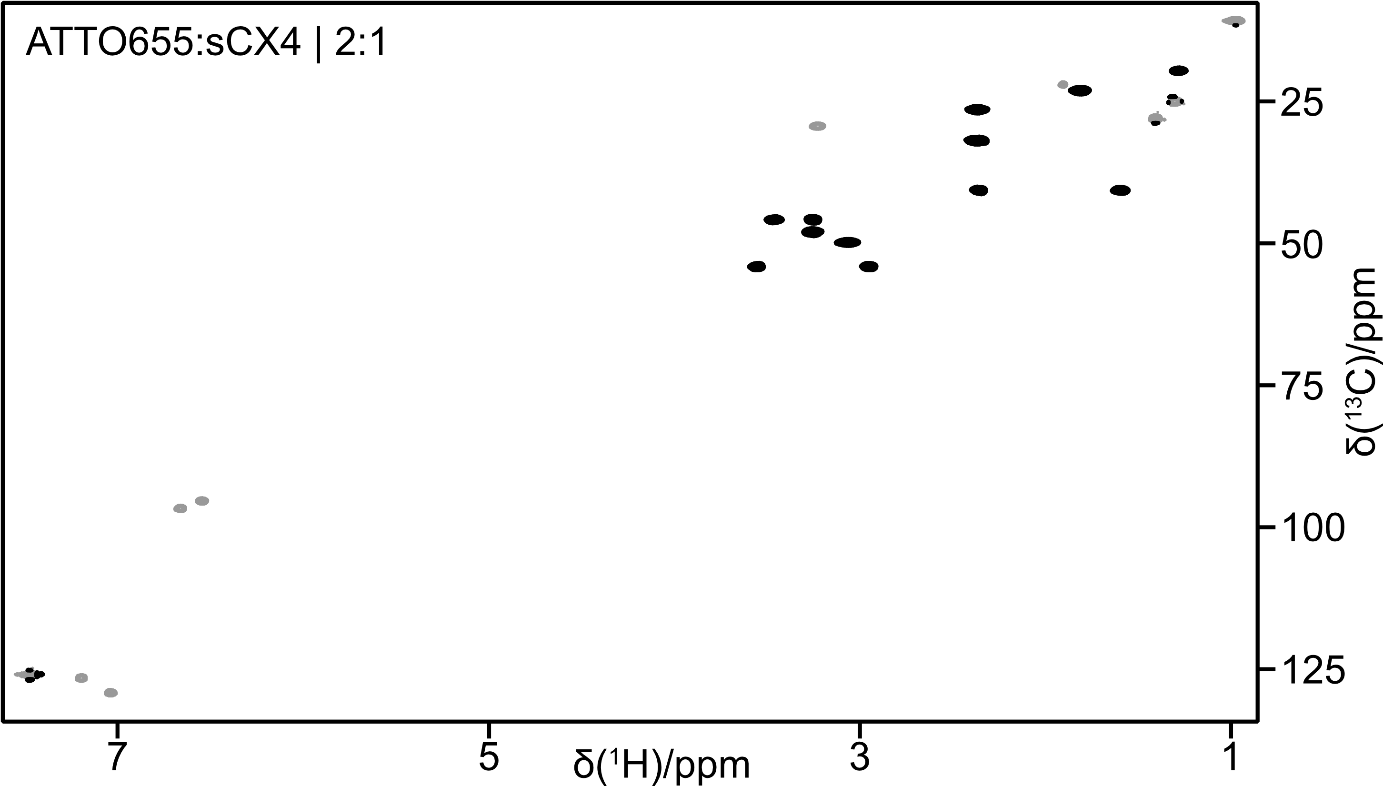


**Figure S31.** 2D ^1^H-^13^C HSQC with multiplicity editing recorded on a ATTO655:sCX4 mixture with 2:1 ratio. Positive amplitude signals are shown in black, negative ones in gray.

**Table S1.** Assignments of ATTO655 NMR chemical shifts to specific atoms for the pure dye and mixture with sCX4. In addition to the naming used in this publication, the numbering generated by the Automated Topology Builder (<https://atb.uq.edu.au/molecule.py?molid=1965133>; archived at https://perma.cc/5UPE-VSE7) and the naming scheme used by CcpNmr3 Analysis Assign are given. Please note that assignments are not stereospecific, which concerns positions 4, 16, 20, 21, and 22.

| Atom Name | Atom Name (ATB) | Atom Name (CCPN3) | Chemical Shift ATTO655 (ppm) | Chemical Shift ATTO655:sCX4 1:1 (ppm) | Chemical Shift ATTO655:sCX4 2:1 (ppm) |
| --- | --- | --- | --- | --- | --- |
| C2 | C18 | C2 | 31.13 | - | 31.95 |
| C3 | C17 | C3 | 22.75 | - | 23.10 |
| C4 | C16 | C4 | 45.76 | - | 45.85 |
| C6 | C9 | C6 | 96.58 | - | 96.78 |
| C9 | C4 | C9 | 95.29 | - | 95.46 |
| C12 | C23 | C12 | 129.40 | - | 129.27 |
| C15 | C12 | C15 | 126.72 | - | 126.66 |
| C17 | C11 | C17 | 29.31 | - | 29.34 |
| C18 | C14 | C18 | 40.58 | - | 40.66 |
| C19 | C15 | C19 | 58.16 | - | 58.16 |
| C20 | C20 | C20 | 24.95 | - | 25.04 |
| C21 | C21 | C21 | 27.91 | - | 27.95 |
| C22 | C22 | C22 | 54.09 | - | 54.13 |
| C23 | C25 | C23 | 26.80 | - | 26.46 |
| C24 | C26 | C24 | 20.11 | - | 19.62 |
| C25 | C27 | C25 | 50.19 | - | 49.86 |
| C26 | C2 | C26 | 48.25 | - | 48.04 |
| C27 | C1 | C27 | 10.93 | - | 10.79 |
| H2a, H2b | H16, H17 | H2% | 2.41 | 2.37 | 2.36 |
| H3a, H3b | H14, H15 | H3% | 1.79 | 1.88 | 1.81 |
| H4a | H12 | H4a | 3.44 | 3.53 | 3.46 |
| H4b | H13 | H4b | 3.23 | 3.34 | 3.25 |
| H6 | H7 | H6 | 6.64 | 6.74 | 6.66 |
| H9 | H6 | H9 | 6.70 | 6.48 | 6.54 |
| H12 | H27 | H12 | 7.10 | 7.07 | 7.04 |
| H15 | H8 | H15 | 7.11 | 7.34 | 7.19 |
| H17 | H9 | H17 | 3.19 | 3.30 | 3.23 |
| H18a | H10 | H18a | 2.34 | 2.36 | 2.35 |
| H18b | H11 | H18b | 1.56 | 1.67 | 1.59 |
| H20a-c | H19, H20, H21 | H20% | 1.30 | 1.32 | 1.30 |
| H21a-c | H22, H23, H24 | H21% | 1.41 | 1.42 | 1.40 |
| H22a | H25 | H22a | 3.47 | 3.65 | 3.55 |
| H22b | H26 | H22b | 2.91 | 3.02 | 2.95 |
| H23a, H23b | H28, H29 | H23% | 2.78 | 2.12 | 2.36 |
| H24a, H24b | H30, H31 | H24% | 1.95 | 0.86 | 1.27 |
| H25a, H25b | H32, H33 | H25% | 3.61 | 2.73 | 3.06 |
| H26a, H26b | H4, H5 | H26% | 3.57 | 3.07 | 3.25 |
| H27a-c | H1, H2, H3 | H27% | 1.25 | 0.77 | 0.97 |

**Geometrical modelling of the ATTO655-sCX4 complex**

All-atom host–guest simulations of ATTO655 sCX4 complexes were performed using the Integrative Modeling Platform[^4^]. The fourfold sCX4 symmetry and the limited number of NMR restraints leave the binding geometry underdetermined. Thus, structural models were determined by IMP’s Bayesian modeling framework. Briefly, atomic coordinates and bond connectivity of optimized structures were represented at full atomic resolution by a custom force field preserving geometry, planarity, excluded volume, and a Go-like native-contact potential; full parameterization details and scripts are available at https://github.com/tpeulen/scx4. NMR restraints between the sCX4 Hα protons and the ATTO655 protons were incorporated in two forms. NOESY-restraints were modeled as asymmetric flat-bottom harmonic upper-bound potentials evaluated against all four equivalent proton pairs with only the closest pair contributing to the score. CSP restraints were modeled as lower-bound potentials applied to all four equivalent pairs simultaneously. Binding was sampled through 50,000 independent simulations, each starting from a random dye placement and combining rigid-body Monte Carlo with Langevin molecular dynamics; only the final configuration per simulation was retained. The ensemble was analyzed by projecting atomic positions onto a three-dimensional voxel grid to yield insertion depth profiles, lateral radial distributions, and dye-plane orientation histograms.

**Supplementary Note 2: Stopped-flow spectroscopy**

To better understand how macrocycle binding affects the fluorescence of individual ATTO655 molecules, we obtained the association (*k*_on_) and dissociation rate constants (*k*_off_) from stopped-flow experiments with fluorescence detection. The apparatus had a deadtime of ~2 ms (Figure S32a), limiting our capability to detect rates slower than 300-500 s^-1^. We monitored both the dissociation and association of ATTO655-CB8 by mixing solutions of the complex with buffer or solutions containing dye and macrocycle, respectively. Importantly, the signal changes for CB8 were consistent with the fluorescence increase upon complex formation (Figure S32b/c).


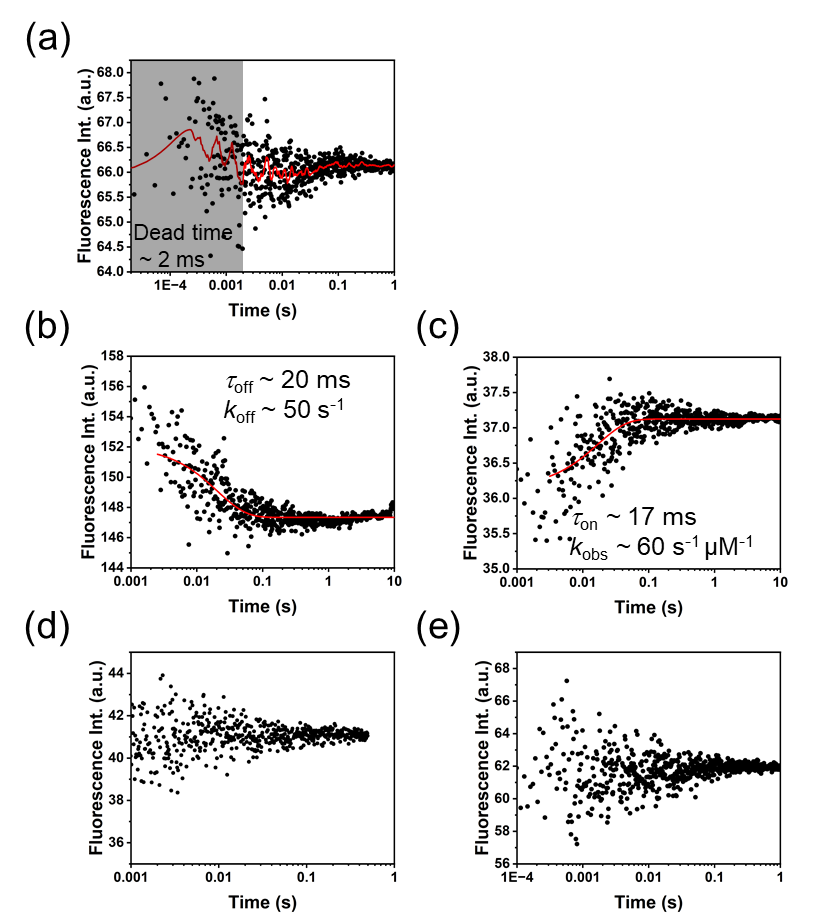


**Figure S32.** Stopped-flow kinetic analysis. Determination of the instrument dead time (~2 ms) performed by mixing ATTO655 with buffer. (b) Dissociation kinetics of the ATTO655-CB8 complex (6.25 µM ATTO655 + 6.25 µM CB8 vs. buffer); τ_-1_ = 20 ms. (c) Association kinetics of ATTO655 and CB8 (ATTO655 1 µM vs. CB8 1 µM); τ_1_ = 17 ms. (d) Dissociation kinetics of the ATTO655-sCX4 complex (10 µM ATTO655 + 2 mM sCX4 vs. buffer) (e) Association kinetics of ATTO655 and sCX4 (5 µM ATTO655 vs. 0.25 mM sCX4).

We found *k*_obs_ values for micromolar concentrations of either ATTO655 or CB8 on the order of 30 – 200 s^-1^ (Figure S32b/c). Assuming pseudo first-order kinetics, which is a very rough assumption due to the nearly equimolar concentrations used, we were able to estimate the order of magnitude of *k*_on_ and *k*_off_ from a linear regression of *k*_obs_ = *k*_on_ ⋅[CB8]_0_ + *k*_off_ (Figure S33). In agreement with data for other complexes, e.g., of CB7 and naphthalene-derivatives[^5^], _kon_ for ATTO655-CB8 was on the order of ~10^7^ M^-1^ s^-1^ with a *k*_off_ ~50 s^-1^. As expected for low-affinity complexes, which are known for association rate constants of 10^8^ M^-1^ s^-1^ or higher, we were unable to detect any signal changes for the association or dissociation reaction of ATTO655-sCX4 (Figure S32d/e). This confirms that the FCS relaxation time of 4.1 µs seen at [sCX4] = 0.5 mM indeed corresponds to complex formation and dissociation (Figures 3g/S7). Based on the relaxation time from FCS and assuming an association rate constant of *k*_on_ = 10^8^ M^-1^ s^-1^, we can estimate the dissociation rate constant of ATTO655-sCX4 to be on the order of ~10^5^ s^-1^, which is in agreement with previous data on pyronine and cyclodextrins[^6^].


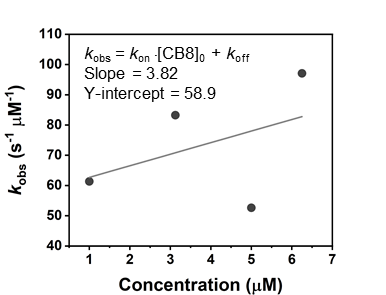


**Figure S33.** Stopped-flow kinetic analysis of the association between CB8 and ATTO655. The plot displays the dependence of the observed pseudo-first-order rate constant (*k*_obs_) on CB8 concentration.

**Supplementary Note 3: Materials and Methods**

**Materials**

ATTO655 (ATTO655 Carboxy) was purchased from ATTO-TEC (Siegen, Germany now Leica, Germany). Cucurbit[8]uril (CB8) and *p*-sulfonatocalix[4]arene (sCX4) were purchased from Sigma-Aldrich and TCI, respectively. The pH of the sCX4 stock solution was adjusted to 7.4 using KOH. Potassium phosphate buffer (100 mM KP_i_, pH 7.4) was prepared from K_2_HPO_4_ (69.6 mM) and KH_2_PO_4_ (30.4 mM). Unless stated otherwise, experimental working solutions contained 5 mM KP_i_ for CB8 and 100 mM KP_i_ for sCX4. Water was purified via a Milli-Q system (Millipore). Functionalized polyethylene glycol (mPEG-silane and biotin-PEG-silane) were purchased from Laysan Bio (USA). Synthetic DNA oligonucleotide (P1-ATTO655: ATTO655-5'-TAA TAT TCG ATT CCT CTG GAC G-3', P2-biotin: biotin-5'-CGT CCA GAG GAA ZCG AAT ATT A-3' -Z: Cy3B-dT) were purchased from Ella Biotech (Germany). Double-stranded DNA with biotin modification, ATTO655-dsDNA, was prepared by hybridizing P1-ATTO655 with P2-biotin using a standard DNA hybridization protocol. Other common chemical reagents (*e.g.* salt and solvent) were purchased from standard commercial suppliers. All materials were used without additional purification.

**Preparation of Immobilized ATTO655-dsDNA**

DNA hybridization. P1-ATTO655 and P2-biotin (both 1 μL, 100 μM) were added to 98 μL of annealing buffer (20 mM Tris-HCl, 500 mM NaCl and 1 mM EDTA in aqueous solution, pH 8.0). The mixture was heated to 95 °C at a rate of 4 °C/s for 5 minutes, followed by gradual cooling to 4 °C at a rate of 0.02 °C/s to yield ATTO655-dsDNA at 1 μM via thermal cycler (Eppendorf Mastercycler X50a). Meanwhile, a biotin-PEGylated cover slip was prepared for immobilization as described below.

Biotin-PEGylated cover slips preparation. Cover slips (24 × 60 mm) were sequentially cleaned with acetone, KOH (1 M) and ethanol, followed by oxygen plasma treatment using a plasma cleaner (Diener electronic Femto low-pressure plasma system) for 20 min. The cleaned cover slips were then immersed into a toluene solution of mPEG-silane (10 mg mL^-1^) and biotin-PEG-silane (0.1 mg mL^-1^) and incubate at 60 °C overnight. After incubation, the biotin-PEGylated coverslips were washed with ethanol and water. They were then mounted onto an 18-microwell sticky chamber (ibidi) and ready for use. ATTO655-dsDNA was immobilized on biotin-PEGylated cover slip via biotin-streptavidin interaction:

Surface immobilization. Bovine serum albumin (BSA, 1 mg mL^-1^ in PBS buffer) was added to the chamber of biotin-PEGylated coverslips and incubated for 10 mins to passivate the surface. Streptavidin (0.2 mg mL^-1^) was then added and incubated for an additional 10 mins. Finally, ATTO655-dsDNA (1 nM) was added and incubated for 30 s to achieve surface immobilization. Each step was followed by a washing process to remove excess reagents.

**UV/VIS Spectroscopy**

Absorbance spectra were recorded using a Shimadzu UV-1800 spectrophotometer with Sarstedt acrylic cuvettes (10 × 10 × 45 mm). Fluorescence spectra were acquired using a JASCO FP-8550 Fluorescence Spectrometer with the same cuvettes. Titrations between the supramolecular host and fluorophore guest were performed by mixing freshly prepared solutions of the host at various concentrations with the fluorophore stock in a 1:1 ratio. The concentration of fluorophore was kept constant to be 1 – 10 μM (absorbance) or 0.1 – 1 μM (fluorescence) within one experimental series. All titrations were performed in independent triplicates (n = 3) If the fluorophore concentration is significantly lower than the dissociation constant (*K*_d_) and the binding stoichiometry is 1:1, the relationship between the total concentration of supramolecular host (*c*) and *K*_d_ is described by one-site binding model, which is expressed as

$$\begin{aligned} y=\frac{c}{K_{d}+c} \#\left( 1 \right) \end{aligned}$$

In this equation, *y* is fraction of bound fluorophore, which can be calculated by the following equation.

$$\begin{aligned} y=\frac{I-I_{0}}{I_{1}-I_{0}} \#\left( 2 \right) \end{aligned}$$

where *I* is the current signal intensity (e.g. fluorescence intensity), *I_0_* initial signal intensity and *I_1_* final signal intensity.

**NMR spectroscopy**

ATTO655, sCX4 and their mixtures were dissolved in heavy water (D_2_O) and the pH adjusted to 7 with deuterated sodium hydroxide (NaOD, 99.5% D purity) purchased from Eurisotop.

Experiments were recorded on a Bruker Ascend^TM^ 800 magnet operating at 800 MHz ^1^H Larmor frequency with an Avance Neo console. A 5 mm, four-channel (^1^H, ^2^H, ^13^C, ^15^N) cryo-probe was used with samples being measured in 5 mm (sCX4) or 3 mm (ATTO655 and ATTO655:sCX4 mixtures) tubes.

Temperature control was achieved with a BCU II from Bruker with the sample temperature adjusted to 25°C. For assignment, the manufacturer-provided pulse sequences *noah4_BSCN* and *noesyfpgpphwg* were used. For the NOESY (*noesyfpgpphwg*) experiment, the mixing time was set to 800 ms and the experiment was run with the LABEL_CN flag. The *noah4_BSCN* pulse program yielded HMBC, HQSC, COSY, and NOESY (mixing time 500 ms) spectra. The EDIT option was used which gave positive signals CH_2_ groups and negative amplitudes for CH and CH_3_ groups in the HSQC experiment.

For chemical-shift perturbation (CSP) calculation, the absolute difference in ^1^H chemical for the two HSQC experiments (ATTO655 alone and ATTO655:sCX4 2:1) was determined. Chemical shifts that changed less than 0.03 ppm (Δδ(^1^H) < 0.03ppm) were considered unaffected and thus remote from the binding interface.

**Stopped-flow experiments**

Kinetic measurements of complex association and dissociation were performed using a KinetAsyst SF-61SX2 stopped-flow system (TgK Scientific) equipped with fluorescence detection. Samples were excited at 646 nm with emission collected through a red band-pass filter (ET685/80M, Chroma). All stopped-flow measurements were conducted at room temperature. The instrument dead time was experimentally determined to be approximately 2 ms based on triplicate control measurements. In these experiments, a solution of ATTO655 was mixed with pure buffer to monitor the non-reactive baseline. The dead time was defined as the time interval between the flow-stop trigger and the stabilization of the fluorescence signal, corresponding to the duration of the hydrodynamic mixing artifact. Association kinetics were monitored by mixing dye solutions with varying concentrations of the macrocycle, while dissociation was measured by mixing the pre-formed complex with buffer. All kinetic traces represent the average of at least three independent injections to improve the signal-to-noise ratio.

**ITC experiments**

ITC experiments were performed using a PEAQ-ITC Automated calorimeter (Malvern Panalytical) to determine the thermodynamic parameters of complex formation between sCX4 and its guests (ATTO655 or choline). All solutions were prepared in 100 mM KP_i_ buffer (pH 7.4), degassed, and thermostated at 20 °C using a ThermoVac sample preparation unit for 15 min prior to loading. For the ATTO655-sCX4 experiment, the sample cell contained 0.72 mM ATTO655, while the syringe was loaded with 12 mM sCX4. For the choline-sCX4 experiment, the sample cell contained 0.4 mM sCX4, and the syringe was loaded with 4 mM choline. Data were processed using the MicroCal PEAQ-ITC Analysis Software. The integrated heat data were fitted to a ‘One Set of Sites’ binding model to yield the *K*_d_, binding stoichiometry (*N*), and enthalpy of binding (∆*H*).

**Microscopy**

One-color excitation (OCE) experiments were conducted on an inverted microscope with time-resolved single photon detection (MicroTime 200, PicoQuant) equipped with single photon counting electronics and picosecond time resolution (Hydra Harp 400, PicoQuant). In OCE experiments, the sample was excited either through a 60x water immersion objective (Nikon M Plan Apo NA 1.20) or a 60x oil immersion objective (Olympus UPlanSApo NA 1.2) to in a diffraction-limited focus. Sample emission was collected through the objective and separated into perpendicular and parallel components using a polarizing beam splitter. Photons were detected by single-photon avalanche diodes (SPCM-AQR-14, Perkin Elmer) after passing through a red (ET700/75M, Chroma) band-pass filter. In OCE, an LDH-D-C-640 (636 nm) linearly polarized pulsed laser diode (PicoQuant) excited the sample at a laser excitation period of 25 ns. Images of were acquired with a Piezo stage (E-725, PI-Instruments) through a the 60x oil immersion objective (Olympus UPlanSApo NA 1.2) with typical image sizes of 256 × 256 pixels (pixel dwell time: 2 ms, pixel size: 40 nm) using a power of 5 µW (1.7 kW cm^-2^) at the objective.

**Fluorescence decay analysis**

Fluorescence decays were recorded by TCSPC on a MicroTime200 (see Microscropy section). The registered photon arrival times were binned into fluorescence decay histograms using the software ChiSurf[^7^] at a typical temporal resolution of 32 ps per bin. The resulting fluorescence decay curves, $f(t)$, were described by multi-exponential models.

$$\begin{aligned} f\left( t \right)=\sum_{i=1}^{N} x^{\left( i \right)}\cdot e^{-\frac{t}{\tau^{\left( i \right)}}}\#\left( 3 \right) \end{aligned}$$

where $x^{(i)}$ and $\tau^{(i)}$ denote the species fractions and fluorescence lifetimes, respectively, and $N$ is the number of fluorescence decay components. Most fluorescence decays were either described by single-exponential or bi-exponential models ($N=2$ or 1). For multi-exponential models we compute $\left\langle\tau\right\rangle_{Amp.}$ and $\left\langle\tau\right\rangle_{Int.}$ as average lifetimes.

$$\begin{aligned} \left\langle\tau\right\rangle_{Amp.}=\sum_{i=1}^{N} x^{\left( i \right)}\cdot\tau^{\left( i \right)}\#\left( 4 \right) \end{aligned}$$

$$\begin{aligned} \left\langle\tau\right\rangle_{Int.}=\sum_{i=1}^{N} x^{\left( i \right)}\cdot{{(\tau}^{\left( i \right)})}^{2}\#\left( 5 \right) \end{aligned}$$

The average $\left\langle\tau\right\rangle_{Amp.}$ represents the species averaged lifetime, while $\left\langle\tau\right\rangle_{Int.}$ represents the fluorescence intesntiy weighted lifetime.

The model parameters were determined by iterative reconvolution fitting, explicitly accounting for the experimentally measured instrument response function (IRF). The IRF was obtained by the scattered light of water under identical optical and detection conditions as the sample. During optimization of the model parameter, experimental nuisance parameters were simultaneously optimized, such as a constant non-fluorescent background contribution (*e.g.*, detector dark counts), a temporal shift between the IRF and the fluorescence decay, and residual scattered light contributions. Parameter optimization was performed by minimizing the reduced chi-square statistic, and goodness of fit was additionally assessed by inspection of weighted residuals.

**Fluorescence correlation spectroscopy**

The registered photon streams were correlated with a multi-tau algorithm[^8^] implemented in tttrlib[^9^] and made accessible in the software Chisurf[^7^]. The computed autocorrelation functions, $G(\tau)$, were described by the product of $G_{d}\left( \tau\right)$, the correlation function for a Brownian diffusion model and $G_{k}\left( \tau\right)$, a kinetic term, that accounts for dark states:

$$\begin{aligned} G\left( \tau\right)=G_{d}\left( \tau\right)\cdot G_{k}\left( \tau\right)+b\#\left( 6 \right) \end{aligned}$$

Above $b$ is a constant offset. $G_{d}\left( \tau\right)$ depends on the effective number of molecules, the correlation time, the diffusion time, and a structure factor of the confocal volume are $N$, $\tau$, $\tau_{d}$, $s$, respectively:

$$\begin{aligned} G_{d}\left( \tau\right)=\frac{1}{N}\cdot\left( 1+\frac{\tau}{\tau_{d}} \right)^{-1}\cdot\left( 1+\frac{1}{s^{2}}\cdot\frac{\tau}{\tau_{d}} \right)^{-0.5}\#\left( 7 \right) \end{aligned}$$

The kinetic term $G_{k}\left( \tau\right)$ was described by:

$$\begin{aligned} G_{k}\left( \tau\right)=\left( 1-\sum_{i=1}^{K} \left[ b_{a,i}+b_{a,i}\cdot\exp\left( -\frac{\tau}{b_{t,i}} \right) \right] \right)\#\left( 8 \right) \end{aligned}$$

Whereas $b_{a,i}$ is an amplitude with corresponding relaxation time $b_{t,i}$ respectively and $K$ is the number of relaxation times. In our experiments $K=1$.

**Pixel and burst-wise spectroscopy**

The used approaches to analyze a group of photons within a burst (burst-wise analysis) or pixel (imaging) are implemented in open-source software[^7, 9^]. Briefly, for every burst or pixels we determine simple spectroscopic parameters, such as integrated photon counts, $n_{X}$, corresponding signal intensities, $S_{X}$[^10^], steady-state anisotropies, $r_{S}$[^11^], average burst pixel arrival times, $T_{X}$, intensity variations in bursts/pixels, and mean fluorescence-weighted lifetimes, $\left\langle\tau\right\rangle_{Int.}$ using a single-exponential re-convolution[^12^].

In the burst-wise single-molecule analysis, we group the photon stream into individual bursts by discriminating the background (approximately 1 – 2 kHz) form the fluorescence signal by applying an intensity threshold criteria[^13^]. In pixel-wise analysis, the time between the line-trigger events defines group photons for pixel. The limited photon number prohibits the accurate determination of complex lifetime models[^14, 15^]. Hence, we determine per burst/pixel an average fluorescence lifetime $\left\langle\tau\right\rangle_{Int.}$ using single-exponential fluorescence decay model without correction of spectral excitation and emission crosstalk convolved with an experimental instrument response function using maximum likelihood estimators[^12^].

**Single-molecule imaging**

Confocal images were reconstructed from TTTR photon data by summing photon counts per pixel over the selected detector channels[^9^].The resulting total-intensity image was smoothed using a Gaussian filter, thresholded (Otsu or fixed), and cleaned of border-touching objects. Individual molecules were identified using distance-transform-based watershed segmentation, with local maxima serving as segmentation seeds. Photons within each segmented region were assigned to individual molecules for subsequent decay analysis as described for burst-wise single-molecule spectroscopy. All image reconstruction, segmentation, and molecule-level data extraction were implemented as a plugin for ChiSurf, enabling direct integration with established TCSPC and fluorescence lifetime analysis workflows.

**SI References**

(1) Ē. Kupče, T. D. W. Claridge. *Chem. Commun.* 2018, **54**, 7139-7142, <https://doi.org/10.1039/C8CC03296C>.

(2) M. Piotto, V. Saudek, V. Sklenář. *J. Biomol. NMR* 1992, **2**, 661-665, <https://doi.org/10.1007/BF02192855>.

(3) S. P. Skinner, R. H. Fogh, W. Boucher, T. J. Ragan, L. G. Mureddu, G. W. Vuister. *J. Biomol. NMR* 2016, **66**, 111-124, <https://doi.org/10.1007/s10858-016-0060-y>.

(4) D. Russel, K. Lasker, B. Webb, J. Velázquez-Muriel, E. Tjioe, D. Schneidman-Duhovny, et al. *PLOS Biol.* 2012, **10**, e1001244, <https://doi.org/10.1371/journal.pbio.1001244>.

(5) S. Akine, Y. Sakata. *Chem. Lett.* 2020, **49**, 428-441, <https://doi.org/10.1246/cl.200017>.

(6) W. Al-Soufi, B. Reija, M. Novo, S. Felekyan, R. Kühnemuth, C. A. M. Seidel. *J. Am. Chem. Soc.* 2005, **127**, 8775-8784, <https://doi.org/10.1021/ja0508976>.

(7) T.-O. Peulen. *Spectrosc. J.* 2025, **3**, 16, <https://doi.org/10.3390/spectroscj3020016>.

(8) S. Felekyan, R. Kühnemuth, V. Kudryavtsev, C. Sandhagen, W. Becker, C. A. M. Seidel. *Rev. Sci. Instrum.* 2005, **76**, 083104, <https://doi.org/10.1063/1.1946088>.

(9) T.-O. Peulen, K. Hemmen, A. Greife, B. M. Webb, S. Felekyan, A. Sali, et al. *Bioinformatics* 2025, **41**, btaf025, <https://doi.org/10.1093/bioinformatics/btaf025>.

(10) E. Sisamakis, A. Valeri, S. Kalinin, P. J. Rothwell, C. A. M. Seidel. Methods Enzymol. 2010, 475,

455-514, https://doi.org/10.1016/S0076-6879(10)75018-7.

(11) J. Schaffer, A. Volkmer, C. Eggeling, V. Subramaniam, G. Striker, C. A. M. Seidel. *J. Phys. Chem.*

*A* 1999, **103**, 331-336, <https://doi.org/10.1021/jp9833597>.

(12) M. Maus, M. Cotlet, J. Hofkens, T. Gensch, F. C. De Schryver, J. Schaffer, et al. *Anal. Chem.* 2001, **73**, 2078-2086, <https://doi.org/10.1021/ac000877g>.

(13) C. Eggeling, S. Berger, L. Brand, J. R. Fries, J. Schaffer, A. Volkmer, et al. *J. Biotechnol.* 2001, **86**, 163-180, <https://doi.org/10.1016/S0168-1656(00)00412-0>.

(14) M. Köllner, J. Wolfrum. *Chem. Phys. Lett.* 1992, **200**, 199-204, <https://doi.org/10.1016/0009-2614(92)87068-Z>.

(15) T.-O. Peulen, O. Opanasyuk, C. A. M. Seidel. *J. Phys. Chem. B* 2017, **121**, 8211-8241, <https://doi.org/10.1021/acs.jpcb.7b03441>.
